# Physical activity and gestational weight gain: a systematic review of observational studies

**DOI:** 10.1186/s12889-022-14324-0

**Published:** 2022-10-21

**Authors:** Virginie Hamann, Philippe Deruelle, Christophe Enaux, Séverine Deguen, Wahida Kihal-Talantikite

**Affiliations:** 1 LIVE UMR 7362 CNRS (Laboratoire Image Ville Environnement), University of Strasbourg, 67000 Strasbourg, France; 2 Department of Maieutics, Maieutics and Health Sciences, University of Medicine, 67000 Strasbourg, France; 3 Gynecology and obstetrics department, Strasbourg University Hospitals, Strasbourg, France; 4 PHARes Population Health trAnslational Research - Inserm CIC 1401 | Bordeaux Population Health Research Center – Bordeaux University, 33000 Bordeaux, France

**Keywords:** Physical activity, Gestational weight gain, Pregnancy, Observational study

## Abstract

**Background:**

Now that excessive weight gain during pregnancy is recognized as leading to complications during pregnancy that affect foetal growth, limiting weight gain during pregnancy has become a public health concern. Our aim was to perform a systematic review to assess whether observational studies reported associations between Physical Activity (PA) and Gestational Weight Gain (GWG). We were particularly interested in whether insufficient PA might be associated with high GWG.

**Methods:**

Using Preferred Reporting Items for Systematic reviews and Meta-Analyses (PRISMA) guidelines, we searched the MEDLINE ® databases for articles published up to February 2020 concerning case-control, cohort, and ecological studies assessing the association between PA during pregnancy and the risk of excessive and/or inadequate GWG.

**Results:**

21 observational studies on the PA of pregnant women were screened. 11 of these focused on excessive GWG, and of these a majority tend to show a significant association between various aspects of PA and excessive GWG. However, the results were more mitigated when it came to rate of GWG: three studies found that neither meeting PA recommendations nor high levels of total PA nor time spent in moderate vigorous physical activity (MVPA) or engaged in sedentary behaviour were associated with weekly GWG, while two others suggested that pregnant women not meeting PA guidelines in late pregnancy did have a higher rate of GWG. Of the seven studies investigating total GWG, only one found no association with PA. All studies suggested an inverse association between PA and total GWG – yet not all studies are statistically significant.

**Conclusion:**

Despite the small number of observational studies selected for our research, our findings support the main international findings, suggesting that active pregnant women gained less weight than inactive women; a lack of PA may therefore contribute to excessive GWG. The limitations of this body of evidence impede the formulation of firm conclusions. Further studies focusing clearly on the general PA assessment classification scheme are called for, to address limitations capable of affecting the strength of association.

**Supplementary Information:**

The online version contains supplementary material available at 10.1186/s12889-022-14324-0.

## Background

Over the past 30 years, there has been an increase in the prevalence of excess weight and obesity among women of childbearing age in industrialized countries. In response to this trend, the Institute of Medicine (IOM) reviewed and updated its (1990) recommendations on weight gain during pregnancy in 2009 [1]. Yet despite these recommendations, GWG has continued to increase in recent years (for instance, almost three-quarters of women now gain weight beyond the guidelines [2]), and limiting this trend has proved challenging.

It is now recognized that excessive weight gain during pregnancy can both promote subsequent obesity and/or increase pre-existing obesity in the mother [3–5]. Excessive weight gain also leads to complications affecting foetal growth during pregnancy, such as gestational diabetes, hypertension and pre-eclampsia [6, 7]. It is also known that infants exposed to excessive GWG or obesity in utero have a 40% higher risk of childhood obesity [8].

Given what is at stake for women and children, reducing weight gain is a public health concern. Several studies have revealed that diet is one determinant of weight gain during pregnancy [9], though numerous studies also suggest that the practice of suitable and regular PA during pregnancy contributes, alongside a balanced food intake, to prevention of excessive weight gain, reduction of the risk of obstetrical pathologies, and a lower risk of pregnancy-related illness [10–14].

Reasons for decreased PA during pregnancy include the physiological changes of pregnancy. These physiological changes may affect the ability to perform sufficient (and recommended) PA. Oxygen demand, heart rate and resting respiratory rate are all increased from as early as the fifth week of pregnancy; these are related to increased blood and stroke volume as well as increased abdominal volume (as a result of increased uterus size). There is also a forward displacement of the centre of gravity, with lumbar hyperlordosis, paravertebral muscle tension, thoracic kyphosis and diastasis of the rectus muscles and ligament hyperlaxity, due to hormonal impregnation [11, 15, 16].

Weight gain also increases stress on the skeleton, joints, ligaments and muscles – and this can further limit PA. In view of these changes, most pregnant women may limit their PA. It seems necessary, then, to adapt PA for pregnant women.

Some studies suggest that the implementation of PA programmes adapted to suit pregnant women have shown their effectiveness at the practice level [11, 14].

In recent years, the number of studies investigating the association between PA and GWG has increased, and the potential impact of PA on GWG has been already reviewed in several meta-analyses based on intervention research or clinical trials [15–17]. These have found that participation in leisure time physical activity (LTPA) is associated with lower weight gain during pregnancy [15]. Overall, physical exercise programmes during pregnancy do lead to a decrease in maternal weight [16, 17].

Yet this research does not lead to a better understanding of the reasons behind spontaneous PA practice by pregnant women in their daily socio-environmental context. Individual behaviour remains at the heart of excessive weight gain prevention, and depends on the empowerment of pregnant women. Observational studies allow measurement (without intervention bias) of the health benefit of the practice of pregnant women’s spontaneous and voluntary daily PA, while also considering their socioeconomic environment.

We aimed to perform a systematic review to assess whether observational studies reporting associations between PA and GWG allow further insights. We were particularly interested in whether PA level, type or other PA characteristics might be associated with high GWG.

To our knowledge, no systematic review aimed at building insight into the relationship between various aspects of PA and GWG has been performed to assess whether observational studies have reported associations between PA during pregnancy and GWG.

In this context, the performance of a literature synthesis may tell us whether the current epidemiological evidence favours an association between PA and GWG, with a view to suggesting future directions and recommendations for research. The aim of this study was to evaluate whether, in the absence of programmed intervention, certain aspects of PA might be associated with various adverse GWG outcomes in observational studies.

## Materials and methods

### Search strategy

Using the PubMed platform, a systematic literature search was conducted – providing access to the MEDLINE databases among articles published up until May 2022. The search strategy followed PRISMA guidelines[18] and was performed using the following keywords in article titles and/or abstracts: (“pregnant women” or “pregnancy”) and (“obese women” or “overweight women” or “gestational weight gain” or “obesity” or “BMI” or “Body Mass Index”) and (“physical activity” or “lifestyle” or “neighbourhood” or “sedentary behaviour” or " physical exercise” or “recreational”).

### Study selection strategy

At the first stage, the inclusion criteria were human studies, peer-reviewed articles written in English and published post-2000. Papers presenting non-original studies or clinical trials or systematic reviews or interventions or activity programmes or other subjects were ultimately excluded. We limited our systematic review to pregnant women and their PA.

At the second step, our exclusion criteria were: *(i)* an absence of assessment of the association between PA types/levels and reported weight gain; *(ii)* a study population limited to overweight or obese women; *(iii)* studies reporting PA and GWG assessment without quantifying the associations between the two.

Using information from titles, abstracts and full manuscripts, the papers were screened independently by two authors (VS and WK) to select those considered relevant, using the screening criteria described below.

At the final step, bibliographic reference lists of all included studies were screened manually to identify additional studies cited by the previous references.

### Data extraction

For each study, we extracted the following information before transferring it into several tables: *(i)* general information: first author’s name, country of origin and date of study; *(ii)* main study characteristics: study design, period, location, statistical methods, population size, main findings (related PA, GWG or rate of GWG); *(iii)* participant characteristics: information on confounders; vi) outcomes (definition, measure, assessment during pregnancy, database); *v)* assessments of association (including odds ratios (ORs), 95% confidence intervals, p-values and other parameters measuring strength of association between PA and GWG). Where several measures of association were available, we reported those from the fully-adjusted models.

The two independent authors (VH and WK) independently extracted all data from selected studies.

## Results

### Studies selected for review

**Fig. 1:**
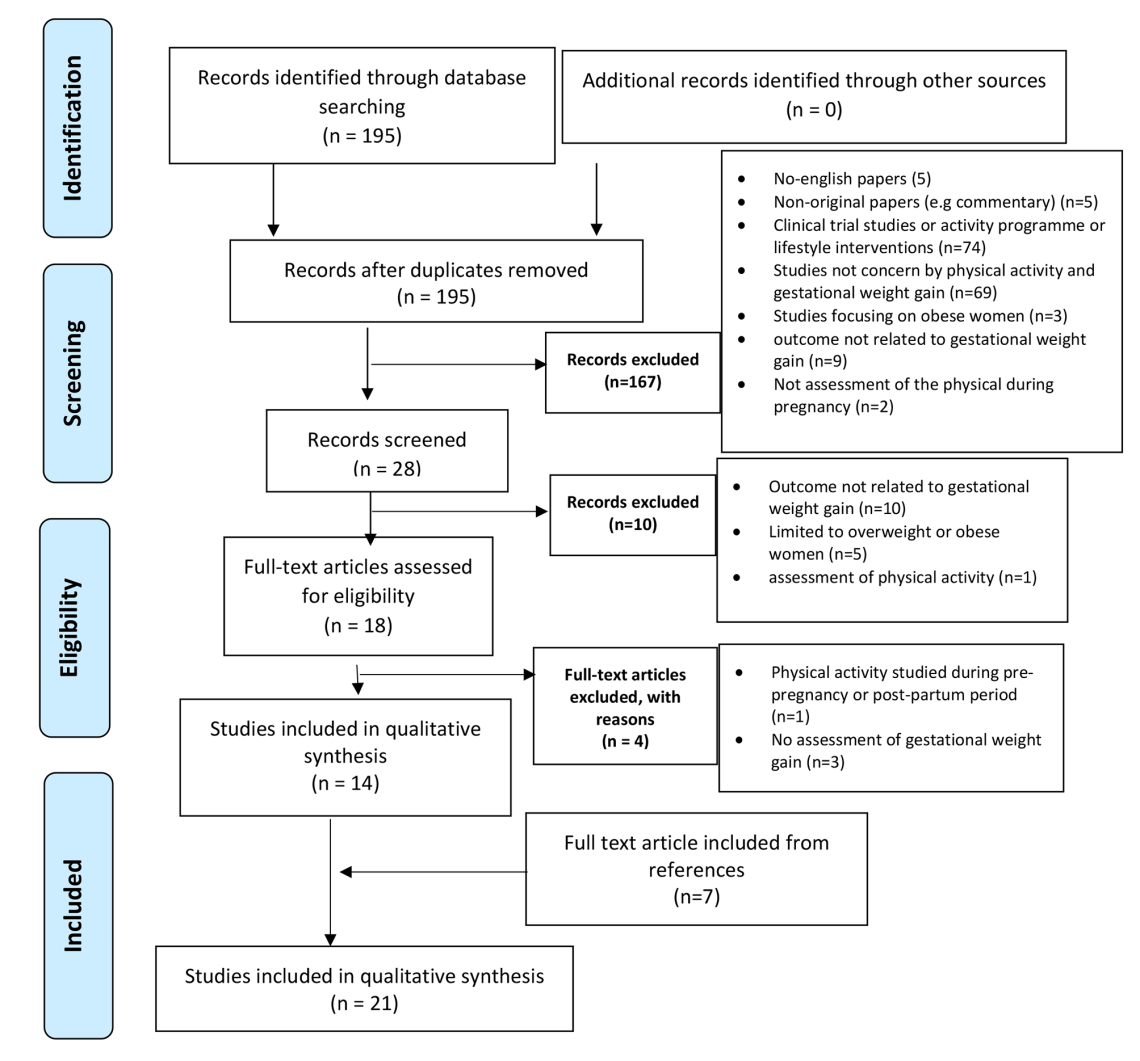
Stages of the selection process PRISMA 2009 Flow Diagram.[18]

In accordance with the criteria summarized in Fig. 1, of the 195 published articles selected, a total of 167 were excluded on the basis of their titles. According to the criteria described above, 27 published articles remained.

In the second stage, the abstracts of these 28 articles were read independently by two authors (VH and WK). This resulted in the exclusion of a further ten studies, based on the criteria described above.

Full manuscripts of the remaining 18 (of the 195 initially selected) articles were read by the two authors (VH and WK). In the end, a further four articles were excluded, in line with our inclusion criteria.

In the last step, bibliographic reference lists of all included studies were searched manually to identify additional studies cited by the previous references. Seven additional articles were included.

In the end, a total of 21 articles met our inclusion criteria for the systematic literature review.

Figure 1 (below) summarizes the various stages of the selection process, in line with PRISMA recommendations.

### General description

Table 1 shows the characteristics of all studies reviewed, organized by year of publication, type of study design, GWG outcome, PA assessment and major findings and conclusions.

**Table 1:** Main characteristics of the selected studies, ordered by year of publication

Authors, Years	Study design, period location	Population size	Outcomes	PA dimensions assessed	Statistical methods	Confounders / stratification	Main findings
Olson et al. 2003 [19]	Cohort study, no information on the period New York (US)	622 pregnant women	Excessive GWG (according 2009 IOM guidelines) Inadequate GWS (according 2009 IOM guidelines)	Self-reported PA: Level PA	Multiple linear and logistic regression model	Maternal characteristics-BMI, the trimester that the prenatal questionnaire was completed, the weeks of gestation, the weeks from the first to the last weight measurement, the weeks from the last measurement to delivery.	Physical activity was significantly related to excessive but not inadequate GWG.
Haakstad et al. 2007 [20]	Cross-sectional survey no information on the period Oslo (Norway)	467 pregnant women	Excessive GWG (According 2009 IOM guidelines) Inadequate GWG (< 16 kg) Overweight (BMI > 25)	Self-reported PA PA level, Sedentary activities, PA duration	The x2-test	Maternal characteristic- none	Women who exercised regularly had lower weight gain than inactive women.
Stuebe et al. 2009 [21]	Cohort study no information on the period Massachusetts (US)	1388 pregnant women	Total GWG Excessive GWG (According 2009 IOM guidelines)	Self-reported PA -PA duration -PA level -Type of PA - Sedentary	Multivariable logistic and linear regression	Maternal characteristic- Pre-pregnancy BMI, age, race/ethnicity, smoking status, gestational age at delivery, and nausea in the first trimester of pregnancy;	Vigorous activity, walking, and total activity during pregnancy were inversely associated with excessive GWG Walking and vigorous activity were also inversely associated with total GWG.
Cohen et al. 2009 [22]	Ad hoc recruitment of pregnant women From August 2008 to December 2008 Ottawa and Montreal (Canada)	81 pregnant women	Achieving recommanded GWG (According 2009 IOM guidelines)	Self-reported PA -PA duration	Univariate logistic regressions	Maternal characteristics: none	The chance for pregnant women to achieved their recommended GWG increase significantly for those who accumulated > 8.5 MET-hr/wk compared to those accumulated < 8.5 MET-hr/wk.
Melzer et al. 2010 [23]	Observational study no information on the period Geneva, (Switzerland)	44 pregnant women	Total GWG	Objective PA measure - PA level	t-test	Maternal characteristic: none	There is no difference between Active and inactive women in term of body weight gain
Abeysena et al. 2011 [24]	Cohort study May 2001 – April 2002 Sri Lanka	580 pregnant women.	Inadequate GWG (< 2009 IOM guidelines)	Self-reported PA - Type of PA	Multivariate logistic regression	Maternal characteristics- Sleeping during 2nd, 3rd or both trimesters, multiparity, sex of newborn, per capita monthly income, Period of gestation, Period of gestation at recruitment, BMI, gestational age, BMI*Sleeping	Standing and walking more than 5 h per day during the second trimester increase the risk of inadequate weight gain during pregnancy.
Hong Jiang et al. 2012 [25]	Cohort study 2005 to 2007 Changzhou, Jiangsu Province, (China)	862 pregnant women	Total GWG Excessive GWG (According 2009 IOM guidelines)	Objective measure of PA - PA level	Multiple linear and logistic regression	Maternal characteristics- Age, educational level, job type, the families’ income, pre-pregnancy BMI, passive tobacco exposure and food energy intake others: gestational age, newborns sex	The GWG decrease among active women compared to the sedentary women during the 2nd and the 3rd trimesters. The risk of excessive GWG decrease significantly among the active women compared to the sedentary women during the 2nd and the 3rd trimester.
Monpetit et al. 2012 [26]	Prospective study From August to December 2008 Ottawa and Montreal, (Canada)	59 pregnant women	Total GWG	Self-reported PA: - PA level Objective PA measure -Daily steps	Hierarchical multiple regression analyses Pearson correlation coefficients	Maternal characteristic- Energy intake Pre-pregnancy BMI	The step is no significant predictor of GWG. no significant correlation between GWG and steps.
Cohen et al. 2013 [27]	Prospective study no information on the period Ottawa and Montreal (Canada)	61 pregnant women	Total GWG Rate of weight gain (kg/week)	Self-reported PA - PA duration - PA intensity - Type of PA - Sedentary Objective PA measure Daily steps	Pearson correlation coefficients, PCA	Maternal characteristics: none	Results suggest that walking and pedometer steps were associated with the rate of GWG
Kraschnewski et al. 2013 [28]	Cohort study From January 2009 to April 2011 Pennsylvania (US)	2603 Pregnant women	Excessive GWG (According 2009 IOM guidelines)	Self-reported PA - PA duration	Multivariable logistic regression	Maternal characteristic- Prepregnancy weight category, age, Race/Ethnicity, Education, Poverty Status, Marital Status, Gestational age at delivery, Smokes Daily	Results show that meeting the physical activity guidelines during pregnancy was significantly associated with a decrease risk of exceeding GWG recommendations.
Restall et al. 2014 [29]	Cohort study From November 2004 and February 2011 Australia, New Zealand, Ireland,	1950 pregnant women	Excessive GWG (According 2009 IOM guidelines)	Self-reported PA Descline exercise during pregnancy	Multivariate logistic regression	Maternal characteristics- Age, BMI, smoke, Mother’s birth weight, Immigrant in past 5 years, fertility treatment, fish or seafood intake, limiting behavior score, sleep Others: Centre	There is a significant increase risk of GWG among women who decreased their level of exercise during pregnancy compared to those who unchanged.
Ruifrok et al., 2014 [30]	Randomized controlled trials analysed as a cohort From 2005 2006 Amsterdam (Netherlands)	111 pregnant women	Rate of Weight gain (kg/week)	Objective PA measure - PA level - Sedentary	Multivariate regression models	Maternal characteristic- BMI, parity, gestational age Others: intervention group	There is no significant association between MVPA or sedentary behavior at 15 weeks with GWG. No significant associations were found for changes in PA and sedentary behavior from 15 to 32–35 weeks of gestation.
Chasan et al. 2014 [31]	Cohort study From 2006 to 2011 Western Massachusetts (US)	1297 pregnant women	-Total GWG - Rate of Weight gain (kg/week) -Inadequate GWG - Excessive GWG (According 2009 IOM guidelines)	Self-reported PA -PA duration -PA intensity - Type of PA - Sedentary - Met PA guidelines	Multinomial logistic regression Linear regression models	Maternal characteristics-pre-pregnancy BMI, age, parity, smoking	There is no significant association between inadequate and excessive GWG and late pregnancy physical activity. However, the total and rate of GWG increase significantly with total physical activity and with physical activity guideline.
Schlaff et al. 2014 [32]	Cohort study From 2008 to2012 Michigan (US)	135 pregnant women	Excessive GWG (According 2009 IOM guidelines)	Self-reported PA - LTPA level	Multivariate logistic regression model	Maternal characteristics: WIC	Results suggest that LTPA level was not significantly related to appropriateness of GWG.
Schlaff et al. 2014 [33]	Cohort study from September 1998 to June 2004 Michigan (US)	449 pregnant women	Inadequate GWG Excessive GWG (According 2009 IOM guidelines)	Self-reported PA -LTPA intensity	Polytomous logistic regression	Maternal characteristics: parity, BMI	Results suggest that LTPA and GWG are not significantly associated.
Merkx et al. 2015 [34]	Cross-sectional survey From September to November 2012 Netherlands	396 pregnant women	Inadequate GWG - Excessive GWG (According 2009 IOM guidelines)	Self-reported PA - motivation healthy PA, - Decline in PA	Multinomial logistic regression	Maternal characteristics- Vegetable consumption, age, gestational age, parity, family income education level, smoking behavior,s atisfied pre-pregnancyweight, perceived BMI	A decline in PA was associated with Excessive GWG.
Ebrahimi et al. 2015 [35]	Cross-sectional study no information on the period Rafsanjan city (Iran)	308 pregnant women	Total GWG Inadequate GWG Excessive GWG (According 2009 IOM guidelines)	Self-reported PA - PA duration - Sedentary	Multivariate Logistic regression models and cumulative logit model	Maternal characteristics- age, education level, and household income, dietary intake, BMI, number of pregnancy.	There is no significant association between PA duration and GWG. Sitting time was positively. associated with gestational weight gain, but the association did not persist in the cumulative logit analysis.
Yong et al. 2016 [36]	Cross-sectional study From November 2010 and April 2012 Selangor and Negeri Sembilan (Malaysia)	589 pregnant women	- Inadequate rate of GWG - Excessive rate of GWG (According 2009 IOM guidelines)	Self-reported PA - PA level	Multinomial logistic regression	Maternal characteristics- age, ethnicity, parity,	Women with low PA level were more likely to have excessive GWG, but the result were no significant.
Collings et al. 2020 [37]	Cohort study From Mars 2007 to December 2010 England	2702 pregnant women A	Total GWG	Self-reported PA - PA level	Multivariate Linear regression	Maternal characteristics- age, gestational age at measurement, socioeconomic status, parity, smoking, alcohol consumption, cafeine intake, sleep quality, use of dietary supplements, early-pregnancy BMI, and the number of weeks between mid- and late- pregnancy weight measurements. Stratified: for white British and Pakistani-origin women, separately	No association was found between PA level and GWG.
Anh Vo Van Ha et al. 2020 [38]	Cohort study From 2015 to 2017 Vietnam	1873 pregnant women	Total GWG	Self-reported PA - PA duration - PA intensity - Sedentary	Multiple linear regression models	Maternal characteristic- age, education, gestational diabetes mellitus, history of health-related problems, total energy intake during pregnancy, parity, employment, gestational age, and pre-pregnancy BMI	Women with high PA level, intensity and household/caregiving activities, and occupational PA have significantly less GWG. Result suggest also women with longer sitting time have significant increase GWG.
Sun et al., 2021 [39]	Cohort study From August 2016 to April 2017 Taiwan	747 pregnant women	Excessive GWG (According 2009 IOM guidelines)	Self-reported PA - Decline in PA	Multivariate logistic regression model	Maternal characteristic- age, Pre-pregnancy BMI	A decline in PA was associated with Excessive GWG.

GWG = gestational weight gain, PA = Physical activity, LTPA = Leisure time physical activity, BMI = body mass index, MET = Metabolic Equivalent of Task, WCI = lower socio-economic status, IOM guidelines = The Institute of Medicine guidelines

21 observational studies on the PA of pregnant women had been conducted since 2000, most of which (16) were published between 2011 and 2020. Combined, these studies included 7,324 pregnant women and sought to estimate the relationship between GWG and various aspects of PA. The aspects investigated were GWG, excessive GWG, inadequate GWG, and rate of GWG (Table 1).

### Study design and location

Most of the studies (9) were conducted in North America (including the US and Canada) [19, 21, 22, 26–28, 31–33]. 5 were conducted in European countries [20, 23, 30, 34, 37], 4 in Asia [24, 25, 36, 38, 39] and just one in Iran [35]. In addition, one study covered three countries –namely Australia, New Zealand and Ireland [29].

Two study designs were represented in our systematic review: most are cohort studies [19, 21–33, 37–39] though four are cross-sectional [20, 34–36].

### Gestational weight gain (GWG) definition and data sources

The relationship between PA and excess gestational weight gain has been investigated for a variety of outcomes. The first category is total GWG [19, 21, 23, 25–28, 30, 31, 37, 38], that is, the difference between pre-pregnancy weight and predelivery weight. The second most investigated category of outcome encompassed excessive GWG [20, 21, 25, 28, 31–35, 38, 39] and inadequate GWG [19, 20, 24, 31, 33–35]. In the third outcome category, the GWG rate was defined as average weekly gain in that trimester [27, 30, 31]. More precisely, the rate of GWG was calculated as total pounds gained divided by gestational age at delivery. For each pre-pregnancy BMI category, the rate of GWG was categorized as inadequate [36] or excessive [22, 29, 36].

Most studies used databases extracted from medical records or obtained from self-reporting questionnaires (see Appendix 1).

### Physical activity (PA)

Most frequently, PA measurement was collected via self-administered questionnaires (see Appendix 2). Some papers investigated the objective measurement of PA, using pedometers [22, 25, 26] or accelerometers [23, 30].

Self-reported PA was assessed mainly through self-administrated questionnaires alongside either short questionnaires containing specific questions [19, 20, 24, 28, 32–34], or validated questionnaires [21, 22, 27, 31, 32, 35–39], including the Pregnancy Physical Activity Questionnaire (PPAQ) [22, 27, 31, 38], the Global Physical Activity Questionnaire (GPAQ) [36], the International Physical Activity Questionnaire (IPAQ) [35, 39], the General Practice Physical Activity Questionnaire (GGPAQ) [37] and the Physical Activity Scale for the Elderly (PASE) [21].

Various aspects of PA were used to analyse and investigate the relationship between PA and GWG including duration [21, 28], intensity [27, 31, 38] total PA [22, 26, 27, 31, 35, 36, 38] and PA level [19–21, 32, 37]. Specific aspects were also investigated as PA declined in the course of pregnancy [29, 34, 39] and PA motivation [34].

Several studies also investigated the relationship between PA type and GWG [20, 21, 24, 27, 31, 33, 38] including: leisure PA [20, 33], walking [21, 24], household/caregiving [27, 31, 38], occupational [27, 31, 38] and transportation-sport exercise [27, 31, 38].

Some studies also analysed the effect of a sedentary lifestyle on GWG [21, 27, 31, 33, 35, 38].

### Confounding factors

Most studies adjusted for maternal characteristics (age, BMI, parity) and unhealthy behaviours such as smoking and dietary intake, with some exceptions [20, 22, 23, 27]. However, adjustment variables also differ between studies.

While some studies included all women regardless of age [20–23, 27, 29, 34, 37, 39], others focused on pregnant women aged 16 to 40, including different age intervals [26, 28, 31, 35, 36]. With the exception of a few studies, most included only nulliparous women. Most authors also chose to include only singleton pregnancies [19–21, 24, 25, 28, 29, 31–33, 35–38]. Many studies took BMI data into account. Some identified BMI as inclusion criteria [28–30, 33], while Ruifrok et al. excluded both overweight and obese patients [30]. The other authors excluded patients where there was insufficient BMI data [23, 28, 29, 33, 35].

### Overview of current evidence on the possible effects of PA on GWG

**Fig. 2:**
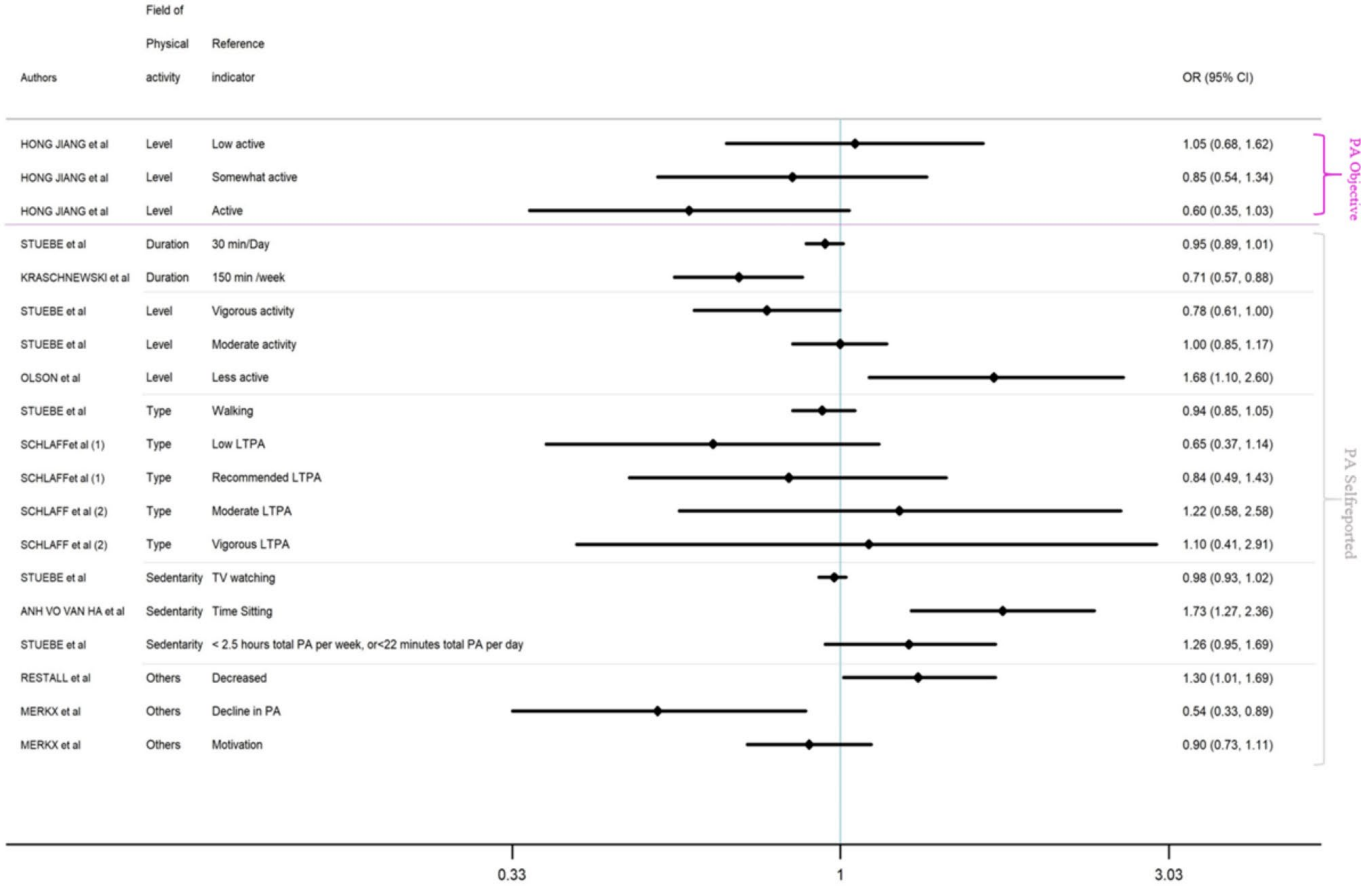
Evidence concerning possible effects on EXCESSIVE GWG of PA

**Fig. 3:**
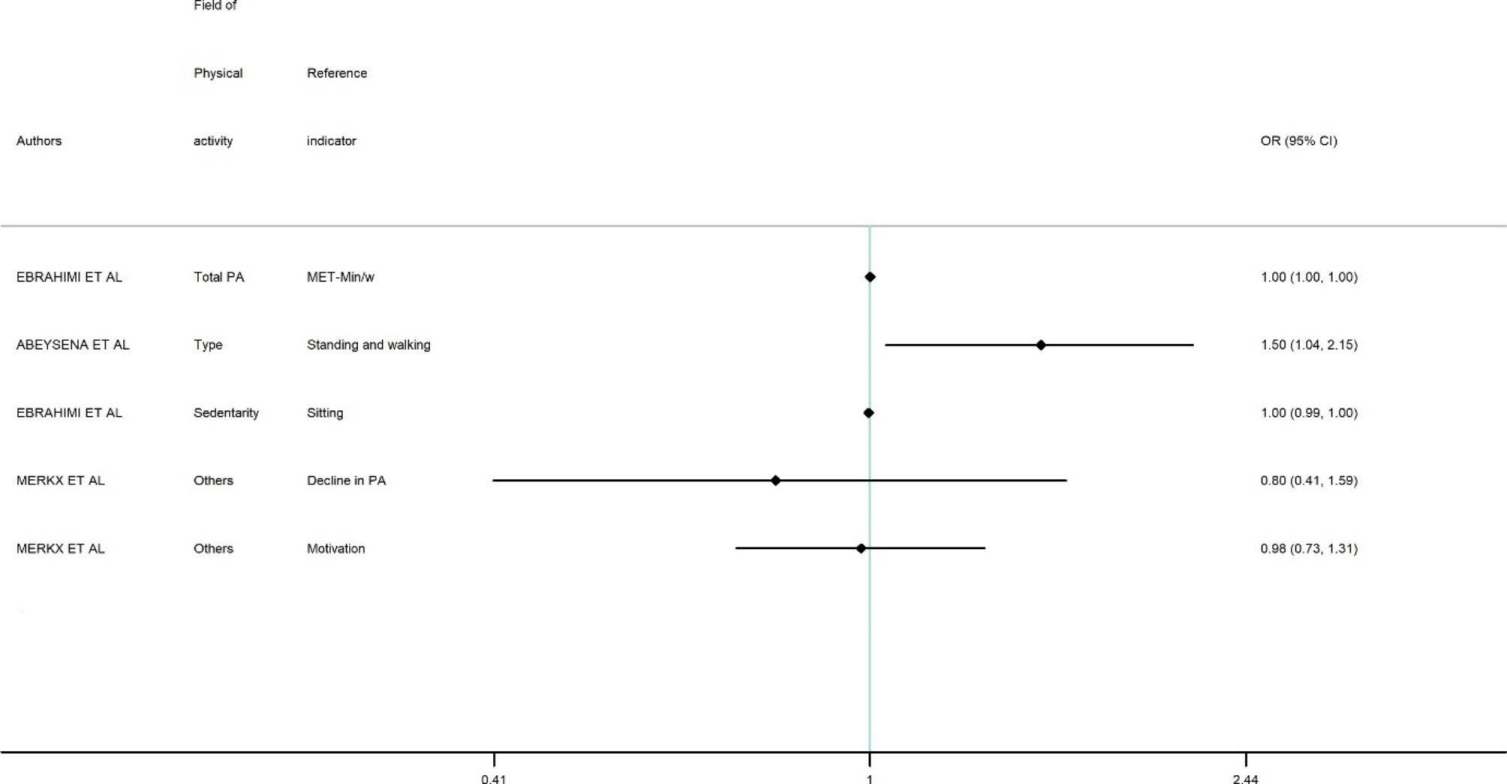
Evidence concerning possible effects on inadequate GWG of PA

In this section, study results were presented in Figs. 2 and 3 and Appendix 3, structured by GWG outcome (Excessive GWG and Inadequate GWG). Overall, results showed that various aspects of PA during pregnancy were significantly related to GWG outcome risks. Nine results tend to show an association between PA and lower risk of excessive GWG [19, 21, 22, 24, 25, 27, 29, 31, 38], while 11 results did not.

### PA and risk of total GWG

Our review revealed that PA type [21, 38], Total PA [21], PA intensity [21, 38], PA level [25, 38] were inversely significantly associated with GWG, while one study found positive association between sedentary behaviour and GWG [38]. For instance, some studies show that walking and total PA decreased the risk of total GWG risk (Beta= -0.25; -0.48 to -0.02 kg per 30 min per day, Beta= -0.48; -1.01 to 0.04 kg per 30 min per day, respectively) [21].

More precisely, some authors found that women with the highest level of moderate-to-vigorous-intensity household/caregiving had a significantly lower total GWG risk (Beta= -0.63 [ -1.11; -0.16]). Ha et al., 2020 found that occupational PA was associated with lower total GWG risk (Beta=-0.79 [-1.35; -0.23]) [38]. In addition, both the highest PA level and a moderate PA level were associated with lower total GWG risk (Beta= -0.37 [-0.90; 0.17] [21]; Beta= -1.45 [-2.44; -0.46] [25] and OR= -0.12; -0.27 to 0.02 kg per 30 min per day [21], respectively).

In particular, PA during the last two trimesters was associated with total GWG. During the third trimester [38] only high PA intensity and a vigorous activity level seem to have any effect on gestational weight [21, 25]. On average, physically active women (having the highest tertile of total PA) gained 0.5 kg less weight during pregnancy than those who were less active [38].

Hong Jiang et al., 2012 [25] found that more active pregnant women had significantly lower maternal weight gain than sedentary women. In the last two trimesters, active women had gained 1.45 kg less than the sedentary group [25]. One study suggested that women with longer sitting time gained 0.6 kg more on average than those who were less sedentary[38].

Some studies revealed no association between PA and total GWG. The authors did however suggest that pregnant women not meeting PA guidelines in late pregnancy had, on average, higher total GWG (3.62 ± 1.48, p = 0.01) compared with those who did meet the guidelines [31].

Some studies observed non-statistically significant association between whether time was spent on MVPA, in sedentary behaviour, on meeting PA recommendations or on high levels of total PA and GWG risk, while other studies were not significant in adjusted analysis [30, 31, 37].

### PA and risk of GWG rate

Conversely, among those studies focusing on the relationship between PA and risk of rate of GWG, results showed that meeting PA recommendations, high levels of total PA, or time spent in MVPA or sedentary behaviour were not found to be associated with weekly GWG [30, 31]. A similar pattern was observed for the risk of an excessive or inadequate rate of GWG [36]. However, Chasan et al. [31] suggested that pregnant women not meeting PA guidelines in late pregnancy had a higher GWG rate (0.08 ± 0.04, p = 0.03) compared with those meeting the guidelines. In addition, Cohen et al. [22] found that if pregnant women had total PA > 8.5 MET-hr/wk were most likely to achieve appropriate GWG (OR = 3.8 [1.18; 12.38]) [22].

### PA and risk of excessive GWG

Among the 11 studies focusing on excessive GWG, a number of results tend to show an association between various aspects of PA and excessive GWG [19, 21, 25, 28, 29, 31–34, 38, 39].

Some studies suggested that self-reported measurement including PA level [19, 21]), sedentary behaviours [21, 38], PA type [21, 31, 32], total PA [21, 28], or a lower level of PA during pregnancy [29, 34] as well as objective PA measurement, including daily steps [25] were related to risk of excessive GWG.

Most studies tended to show that insufficient PA or a sedentary lifestyle were related to increased risk of excessive GWG, though not all are statistically significant.

Our review showed that low PA level and declines in PA levels > 4000 METs-Min/week were positively associated with excessive GWG risk (OR = 1.68 [1.1, 2.6] [19], OR = 2.83 [1.27–4.43] [39], respectively) while both total PA and walking were negatively associated with excessive GWG risk (OR = 0.95 [0.89–1.01] per 30 min per day; OR = 0.92 [0.83–1.01] per 30 min per day, respectively [21]). More precisely, some authors found that meeting the PA guidelines was negatively associated with excessive GWG risk (OR = 0.71 [0.57–0.88]) [28].

In addition, a lower PA level from 14 to 16 weeks, or during pregnancy, was significantly associated with excessive GWG (OR = 1.30 [1.01;1.69]) [29] (OR = 0.54 [0.33;0.89]) [34], respectively.

More precisely, sedentary behaviour such as time spent sitting during pregnancy: (OR = 1.73 [1.27–2.36]) [38] or PA levels of less than 2.5 h per week in total (OR = 1.26 [0.95–1.69]) [21] were associated with increased risk of excessive weight gain.

Among those studies investigating PA by trimester of pregnancy, results tend to show an association between PA and risk of excessive GWG, mainly in the second and third trimesters.

Some studies revealed that PA level during the second trimester was inversely associated with the risk of excessive GWG. Jiang et al., 2012 suggest that for women exceeding the recommended level (more than 10,000 steps per day) the OR was equal to 0.59 [0.36–0.95] [25]. More precisely, Stuebe found that both mid-pregnancy walking (OR = 0.92 [0.83–1.01], per 30 min per day [21]) and vigorous PA in mid-pregnancy (OR = 0.76 [0.60–0.97]) per 30 min per day [21] ) were inversely associated with the risk of excessive GWG.

Other studies suggest that PA level during the third trimester for somewhat-active women (around 7500 ~ 10,000 daily steps) was associated with risk of excessive GWG (OR = 0.66 [0.43- 1.00]) [25] ).

Some studies observed non-statistically significant associations between LTPA [32, 33] or type or intensity of PA during pre, early, mid, or late pregnancy [31] or sedentary behaviours[35] and risk of excessive GWG. However, Chasan et al., 2014 [31] suggested that in comparison with women in the lowest quartile of total PA, women with the highest levels of total PA during early, mid and late pregnancy were not at significantly increased risk of excessive GWG (OR = 1.24 [0.74–2.06]; OR = 1.22 [0.74–2.06]; OR = 0.73 [0.44–1.22] respectively).

### Risk of inadequate GWG and PA

Conversely, among studies focusing on inadequate GWG [19, 24, 31, 33–35], our review showed that three results tend to show an association between PA and the risk of inadequate GWG [24, 31, 35], though not all of these are statistically significant. Three studies found no significant association between PA during pregnancy and inadequate GWG [19, 25, 33].

Abeysena’s study found that women whose standing and walking time was > 5 h per day during the second trimester had a high risk of inadequate GWG (OR = 1.50 [1.04, 2.15]) [24]. Ebrahimi’s study suggested that, women who spent less time sitting had inadequate weight gain in comparison with the adequate GWG group (OR = 0.997 [0.994–0.999]) [35].

Chasan et al., 2014 [31] suggested that in comparison with unemployed women, women having the highest levels of occupational activity were less likely to have inadequate GWG (OR = 0.50 [0.30–0.84]).

In addition, these authors suggested that in comparison with women in the lowest quartile of total PA, women having the highest levels of total PA during early, mid and late pregnancy were not at significantly increased risk of inadequate GWG (OR = 0.98 [0.55–1.73]; OR = 1.06 [0.60–1.80]; OR = 0.73 [0.38–1.40] respectively) [31].

## Discussion

### Main findings

Based on observational studies, while our systematic review tends to show a relationship between PA and excessive GWG, not all studies are statistically significant (see Appendix 3, Figs. 2 and 3).

In addition, our systematic review reveals that various aspects of PA during pregnancy, (especially low PA levels and sedentary behaviours) are related to the risk of excessive GWG. Despite several non-significant associations, most studies suggested that active pregnant women have a lower risk of excessive GWG in comparison with inactive pregnant women.

Our literature review highlights various findings of the studies that could be partially explained by methodological limitations: heterogeneity of PA assessment method, definition of GWG outcome, definition of confounders and statistical approaches.

In addition, several inaccuracies and biases inherent to different analysis methods may bias cross-study comparisons and conclusions drawn from them. These limitations will be discussed below.

### GWG assessment

To fully interpret the findings of the studies, it is important to pay careful attention to GWG assessment, which could constitute a source of uncertainty. We identified three pathways in which outcome information may suffer as a result of uncertainties: (i) method of expressing GWG, (ii) gestational period during which GWG is estimated, and (iii) methods of designating pre-pregnancy weight.

First, different methods of GWG expression have been used, with the most common expression of total GWG being defined as the difference between pre-pregnancy weight and predelivery weight [19–21, 23–28, 30–35, 37, 38], though others defined the GWG rate as weekly GWG [22, 27, 29–31, 36]. In addition, many studies investigated excess GWG as total GWG exceeding *IOM* guidelines, while others defined it as weight gain of more than 15 kg [38] or 16 kg [20]. These different approaches to GWG assessment may lead to difficulty in comparisons between studies.

Second, the use of various gestational periods to estimate GWG may result in substantial misclassification of GWG. Some studies estimate GWG by calculating the difference between predelivery weight and pre-pregnancy weight [19, 21, 23, 26–28, 32–35, 38], while others estimate GWG as the difference between predelivery weight and first trimester weight [24, 25, 31] or between third trimester weight and pre-pregnancy weight [20, 37], or between third trimester weight and first trimester weight [29, 30]. Thus, the length of the period during which weight changes are differently recorded between studies varies, and this may result in underestimation of the true GWG.

Thirdly, most studies estimated GWG on the basis of women’s self-reporting [19–22, 25, 26, 28, 30, 33, 34, 38], and few used data derived from medical measurement [23, 24, 27, 29, 36, 37]. In most studies, with the notable exception of 3 [26, 28, 34], late pregnancy weight (predelivery or pre-birth weight) was extracted from a medical measure. Women tend to under-report their weight prior to pregnancy, when compared with objective measures [40], and this could introduce bias to those results that include pre-pregnancy weight. This would suggest even greater rates of excessive GWG than demonstrated. GWG based on self-reported information may thus result in overestimation by self-reporting, or underestimation if based on late first trimester weight. However, in those studies that have compared self-reporting and medical measurement, the overall pattern of associations remains unchanged [41].

### Confounding factors

The different adjustment factors used in each study may lead to difficulty in summarizing the data. Where no adequate adjustment was performed, it is likely that the strength of the relationship between PA and GWG has been confounded by these factors. For instance, maternal age is an important confounding factor. Some studies suggested that older women showed significantly lower mean GWG than younger women [42, 43]. Pre-pregnancy BMI is known to have a significant effect on GWG: total GWG has been reported to be lower on average in women with high BMI. In addition, there is evidence that smoking is inversely associated with GWG.

Some studies suggest that parity is also known to have a significant impact on GWG [44]. The authors found that multigravid women with high BMI gained less weight than primigravid women with a high BMI, whereas primigravid women with a high BMI gained a lot more weight than primi- and multi- gravid women with medium or low BMI.

### PA assessment

Our systematic review revealed that several approaches to assessing various aspects of PA during pregnancy have been implemented, which could also affect the findings interpretation and thus the accuracy of the conclusion.

Therefore, the limitation of the studies reviewed in the present study lying in PA assessment include (i) factors influencing PA measure (database and information collection), and ii) categorization of PA measure; the association between PA and GWG found in the selected studies may depend on the precision inherent to classification approach of the PA measure chosen in each study.

First, most studies used a validated, self-administered questionnaire including PPAQ, GPAQ, IPAQ, though others used a short, non-validated questionnaire [19, 20, 24, 28]. Only five studies used the same PPAQ questionnaire [22, 26, 27, 31, 38], validated for use with pregnant women. However, self-reported PA overestimates activity in comparison with objective measures. In addition, most questionnaires show poor validity in pregnancy. According to recent meta-analysis, accelerometer measurements are more accurate for PA measurement [45].

Second, the categorization of different PA aspects varied between studies, even among those studies using the same questionnaire. This may alter the findings comparison and lead to misclassification of PA level. Sattler et al. have already highlighted this source of heterogeneity in data collection and PA assessment [41], advocating the development of standards for the use and analysis of PA for future studies. For instance, different criteria were used to define whether pregnant women met the total PA guideline: either accumulation of more than 8.5 MET-hr/week [22, 26, 27], or accumulation of more than 7.5 MET-hr/week in any moderate-intensity or higher activity (30 min/d of activity at ≥ 3 METs multiplied by 5 d/w)[31]. Other studies chose to divide pregnant women’s total PA into quartiles [31] or tertiles [38]. Despite using the same data collection tool, no study used the same categorization for PA types [21, 27, 31, 38]. In terms of PA intensity, with the exception of one study, authors used a homogeneous approach of METs/hours/week [27, 31, 38]. To characterize sedentary behaviours, in 2012 the Sedentary Behaviour Research Network suggested standardization of the term sedentary as any waking behaviour characterized by energy expenditure ≤ 1.5 METs while sitting or lying down [46]. In our review, only four studies used this definition of sedentary behaviour [27, 31, 35, 38]. Other authors used the term ‘inactivity’ to describe the behaviours of women performing insufficient amounts of PA according to specified international recommendations [46]. They used different criteria to define inactive pregnant women having sedentary lifestyles [21, 25, 30, 33] – for instance, LTPA < 7.5 kcal/kg/week [33] or total PA < 2.5 h per week, or total PA < 22 min per day [21], or daily steps < 5000 daily steps [25, 30].

Third, the gestational period during which PA was estimated could induce PA level misclassifications. In our systematic review, different approaches define the gestational period used to investigate the relationship between PA and GWG outcomes: PA throughout the pregnancy, as against during specific periods of pregnancy. Most studies investigated PA throughout the pregnancy [19, 29, 30, 32–34, 38, 39], even where they also performed multiple measurements [22, 24, 27]. Some studies focus on a specific period, with authors measuring and analysing PA separately for each of the three trimesters [20, 31], while others investigated PA during the second [21, 26] or third trimester [23, 28, 35, 37]. The loss of precision inherent to such a general classification scheme of PA reduces the likelihood of detecting an association between PA and GWG. For instance, the time evolution of physiological changes throughout the different trimesters should be considered in PA measurement, limiting analysis to a single trimester rules out precise analysis. In addition, a lack of homogeneity in PA assessment may influence results, and a lack of significant effect for some PA domains may occur.

### Assessment of the relationship between GWG outcome and PA

First, various statistical models were performed to measure the association between PA and GWG. Differences between the statistical methods implemented may obscure interpretation of the results. For instance, most studies used multivariate analysis to analyse the association between PA during pregnancy and GWG (with the exception of four studies [20, 23, 27]), which quantified the correlation using, for instance, a crude Pearson’s correlation coefficient [20, 27]. Many studies used the regression model. Only three authors performed a multinomial analysis [31, 34, 36], while one other study performed a hierarchical model [26].

Second, the sample size of any epidemiological study may affect its statistical power and show an absence of significant association due to lack of power. Our review includes various sample sizes, from the very small (44 pregnant women [23]) to the very large (2,702 pregnant women [37]). Many studies investigated over 1,000 pregnant women [1297; 2702] [22, 24, 29, 30, 32, 39], while a few had sample sizes of between 500 and 1,000 [580; 862] [19, 24, 25, 36], and two had sample sizes of below 100 [44; 81].

### Limitations and risk estimation

All the features of the studies described above – such as study population, study design, sample size, PA assessment and GWG case definition, database used, confounding factors and statistical methods – could (independently or in combination) impact both the results of each study and their comparison in our systematic review. Some factors may result in overestimation of the risk of GWG outcome, while others may result in its underestimation. The lack of precision inherent to such a general classification scheme (definition of outcome) may reduce the likelihood of detecting an association between PA during pregnancy and excessive GWG.

In addition, the various confounding factors included in the individual studies make summarizing the data difficult. An absence of systematic adjustment for commonly-known factors may affect measurement of association, and thus comparisons of all risk estimates—for instance, BMI, age, or the presence of comorbidities known to be related to both GWG and PA.

Self-reported PA may not reflect pregnant women’s actual PA. In addition, PA data was collected during a single period of pregnancy and then assigned to the pregnancy as a whole, rather than in the course of each trimester. This can have a particular impact on studies seeking to explore the risk of GWG outcome.

## Public health implications

Numerous meta-analyses have demonstrated the role of PA in GWG. US studies in particular have shown that excess weight gain can be limited by recourse to a suitable diet combined with vigorous PA and 30 min of walking per day, from the second trimester onwards [47].

Beyond its effects on GWG, PA during pregnancy has beneficial effects for the general health of the pregnant woman. PA allows for a reduction in feelings of fatigue, better postural support, shorter labour and less frequent childbirth complications, a lower risk of depression and improved mood [48–50].

PA also has beneficial effects on subsequent child health. One prospective study showed that the mother’s level of PA during pregnancy was negatively associated with the child’s BMI and waist circumference at age 7, while two other studies found a favourable association at 2 and 5 years of age with language development and certain features of neurological development [51].

A meta-analysis of randomised controlled trials found a 39% reduction in the chances of having a baby > 4000 g (OR 0.61, 95% CI 0.41 to 0.92) in women who exercised compared to those who did not, without affecting the chances of stunted growth, premature growth or low birth weight [52].

When women participate in a suitable PA programme, the effects on both their health and that of their child are proven. Indeed, one study observed 514 three-year-old children, 49% of whose mothers had benefited from an intervention offering PA and 51% from standard care. There was no difference in the main assessment criterion (thickness of the sub-scapular skin fold, between the trial arms − 0.30 mm, IC 95% [-0.92, 0.31]). However, the intervention was associated with a lower resting pulse rate (-5 bpm IC 95% [-8.41, -1.07]) and a lower non-significant probability of overweight/obesity (OR 0.73; 0.50, 1.08) [53].

However, because not all women are able to benefit from this type of intervention, it is essential to promote women’s spontaneous PA during pregnancy, in line with all international and national recommendations [54–56].

In our review, most studies also show that pregnant women’s PA decreases in both duration and intensity during pregnancy. This can be explained by barriers to the practice of PA, such as fear of negative consequences for the child or pregnancy and very poor communication from caregivers about the benefits and details of PA practices during pregnancy [57]. Perceived behavioural control during pregnancy may influence intention to engage in PA [58].

### Future research

Based on the limitation analysis of the current body of research and of theoretical and methodological considerations, we are putting forward some suggestions for improvements and for putting this research on the agenda.

First, further studies are called for, and these should focus clearly on the general classification scheme of PA assessment, so as to resolve limitations capable of affecting strength of association, such as PA assessment and categorization and PA assessment windows during pregnancy. Emphasis could be placed on use of a standard questionnaire to investigate a standard PA assessment, such as meeting PA recommendations.

Second, in future studies, emphasis could be placed on the definition of excess GWG as total GWG exceeding the IOM guideline, which is acknowledged to be more appropriate to the investigation of GWG. Studies would therefore produce more comparable results.

## Conclusion

Despite the small number of epidemiological studies selected in our systematic review, our results tend to show a relationship between excessive GWG and different PA domains, including PA intensity, level and type and sedentary behaviours – yet not all are statistically significant.

Because this body of evidence has limitations that impede the formulation of firm conclusions, new, clearly-focused observational studies are called for.

To provide high quality evidence, future studies must homogenize criteria for PA measurement, categorization and analysis in line with international recommendations specific to pregnant women.

Beyond the recognized overall effects on pregnant women’s health, it seems that pregnant women who failed to meet PA guidelines had, on average, lower total PA than those who did meet the guidelines. Motivation to engage in PA also appears to be associated with a decreased risk of excess GWG. Gaining an understanding of the factors influencing PA among pregnant women is therefore essential to promoting such an understanding, in the absence of an appropriate PA programme. Beyond women’s individual characteristics, an understanding of both socioeconomic and environmental factors also seems an essential perinatal health issue for the prevention and reduction of sedentary behaviours and inactivity in pregnant women.

## Electronic supplementary material

Below is the link to the electronic supplementary material.

Supplementary Material 1

Supplementary Material 2

Supplementary Material 3

Supplementary Material 4

## Data Availability

All data generated or analyzed during this study are included in this published article and its supplementary information files.

## Abbreviations

GWG: gestational weight gain.
PA: Physical activity.
LTPA: Leisure time physical activity.
BMI: body mass index.
MET: Metabolic Equivalent of Task.
WCI: lower socio-economic status.
IOM guidelines: The Institute of Medicine guidelines.
PPAQ: Pregnancy Physical Activity Questionnaire.
GPAQ: Global Physical Activity Questionnaire.
IPAQ: International Physical Activity Questionnaire.
GGPAQ: General Practice Physical Activity Questionnaire.
PASE: Physical Activity Scale for the Elderly.
