# Supplementary material for: Physical activity and gestational weight gain: a systematic review of observational studies
Source: BMC Public Health. 2022 Oct 21;22:1951. doi: 10.1186/s12889-022-14324-0 (PMC9585865; doi:10.1186/s12889-022-14324-0)
Supplement: Supplementary file 1 — Supplementary Material 1 [file 12889_2022_14324_MOESM1_ESM.docx]

**Appendix 1 Table 2:** Definitions of GWG and studied population

| Type(s) or subtype(s) | | Measure/Gestational period | Population study (inclusion and exclusion of pregnant women) | Database of Gestational weight measure | Authors, date |
| --- | --- | --- | --- | --- | --- |
| Total gestational weight gain  (Kg) | **The continuous outcome variable GWG** | | | | |
|  | GWG calculated by subtracting the weight in early pregnancy and late pregnancy | GWG calculated by subtracting the pre-pregnancy weight from the predelivery weight | **Exclusion Criteria** : Heart disease or treatment that may alter cardiovascular conditioning, preeclampsia, diabetes, risk for premature delivery, high probability of cesarean section, fetal malformation or growth retardation delivered before prelabor incomplete measurements. | **Early pregnancy weight:** measured Pre-pregnancy weight.  **Late pregnancy weight:** measured Predelivery weight | Melzer, et al  2010  [25] |
|  |  |  | **Inclusions criteria**: age 18–40 years, Women with second or third trimester free of contraindications to exercise during pregnancy | **Early pregnancy weight:**  self-reported Predelivery weight  **Late pregnancy weight:**  self-reported Predelivery weight | Monpetit et al  2012  [28] |
|  |  |  | **Inclusions criteria:** age ≥18 years, singleton pregnancy | **Early pregnancy weight:**  Self-reported Pre-pregnancy weight  **Late pregnancy weight:**  Pre-birth weight (before delivery) extracted from medical records | Anh Vo Van Ha et al., 2020  [40] |
|  |  |  | **Inclusions** criteria: ≤ 22 weeks gestation at study entry, singleton pregnancy, delivered after 34 weeks | **Early pregnancy weight:**  self-reported Pre-pregnancy weight  **Late pregnancy weigh**t:  Pre-birth weight (before delivery) extracted from medical records | Stuebe et al., 2009  [23] |
|  |  | Late-pregnancy GWG calculated as weight measured at recruitment subtracted from weight in the third trimester (36 weeks) | **Inclusion criteria**: ethnic groups Pakistani-origin and white British Women, singleton birth, free pre-existing hypertension, and diabetes prior to pregnancy  Exclusion criteria: Women from other South Asian ethnicities, including Indian and Bangladeshi or other white backgrounds and mixed ancestry were excluded due to small numbers. | **Early pregnancy weight**: measured Pre-pregnancy weight  **Late pregnancy weight:**  Pre-birth weight (before delivery) extracted from medical records | Collings et al.,  2020  [39] |
|  |  | GWG was calculate as the difference between the first (around 15 weeks of gestation) and the last measurements (at 32 or 35 weeks) | **Inclusions criteria**: Pregnant women nulliparous pregnant women without BMI restrictions or BMI of >25 kg/m2 and singleton pregnancy*  **Exclusion criteria**: Recruitment after 20 weeks, age < 18 years, inadequate knowledge of the Dutch language, gestational diabetes mellitus before randomization, severe chronic disease | **Early pregnancy weight**:  self-reported Pre-pregnancy weight  **Late pregnancy weight:** measured Predelivery weight | Ruifrok et al., 2014  [32] |
|  |  | GWG was calculating the difference between pre-pregnancy weight during first trimester and the pre-birth weight. | **Inclusions criteria**: age≥ 20 years, singleton pregnancy  **Exclusion criteria**: gestational diabetes mellitus, hypertension heart disease, chronic renal disease, other diseases restricting physical activity. | **Early pregnancy weight**:  self-reported Pre-pregnancy weight  **Late pregnancy weight:**  measured Predelivery weight | Hong Jiang et al  2012  [27] |
|  |  | GWG calculated as by subtracting the antenatal weight form the pre-pregnancy weight in the ﬁrst prenatal visit before the 9 or the 11 weeks. | **Inclusion criteria**: Singleton pregnancy, Prenatal care before the third trimester, age ≥18 years at the time of delivery, planned to deliver within the local hospital and keep the baby, Healthy and mentally competent. | **Early pregnancy weight:**  self-reported Pre-pregnancy weight  **Late pregnancy weight:** measured Predelivery weight | Olson et al., 2003  [21] |
|  |  | GWG were self-reporting by women during the 1-month postpartum survey | **Inclusions criteria**: singleton pregnancy, age 18-35 years, nulliparous.  **Exclusion criteria**: women who underweight (BMI <18.5 kg/m2 prior to pregnancy) and delivered prior to 37 weeks gestation, not provide information on height, pre-pregnancy weight or GWG. | Gestational weight gain:  Self-reported (baseline survey) | Kraschnewski et al.,  2013  [30] |
|  |  | Total gestational weight gain: Recorded at by physician at their routine 6 weeks post-partum visit | **Inclusions criteria**: their second and third trimesters without contraindications to exercise as per the PARMed-X for Pregnancy | **Early pregnancy weight**:  self-reported Pre-pregnancy weight  **Late pregnancy weight:** weight extracted from medical records (post-partum visit) | Cohen et al., 2013  (10) |
|  |  | GWG was defined as the difference between maternal weight at delivery and maternal pre-pregnancy weight up to 20 gestational weeks. | **Exclusion criteria**: current medications thought to adversely influence glucose tolerance, multiple gestation, history of diagnosis of diabetes, hypertension, heart disease or chronic renal disease, age <16 years or > 40 years | **Early pregnancy weight**:  self-reported Pre-pregnancy weight or extracted from medical records  **Late pregnancy weight:**  Pre-birth weight (before delivery) extracted from medical records | Chasan et al., 2014  [33] |
| Dichotomized outcome variable GWG | | | | | |
| Excessive GWG | Excessive GWG  (exceeding IOM recommendations) | GWG was calculating the difference between pre-pregnancy weight and the last weight (before delivery) | **Inclusions criteria**: Pregnant women with ≤ 22 weeks gestation at study entry, singleton pregnancy, delivered after 34 weeks  **Exclusion criteria**: women who had a pre-pregnancy BMI classification of underweight | **Early pregnancy weight**:  self-reported Pre-pregnancy weight  **Late pregnancy weigh**t:  Pre-birth weight (before delivery) extracted from medical records | Stuebe et al., 2009  [23] |
|  |  |  | **Inclusions criteria:** age ≤18 years with < 14 weeks gestation at study entry and who delivered a live, singleton infant at term (37 weeks)  **Exclusion criteria**: women who had a pre-pregnancy BMI classification of underweight | **Early pregnancy weight**:  self-reported Pre-pregnancy weight or abstracted from birth certificates  **Late pregnancy weigh**t:  Pre-birth weight (before delivery) extracted from birth certificates | Schlaff et al., 2014  [34] |
|  |  |  | **Inclusions criteria**: age ≥ 15 years, with 6th–27th weeks of pregnancy, singleton pregnancy with no known chromosomal abnormality or birth defect, and no pre-pregnancy diabetes mellitus, maternal serum alpha-fetoprotein (MSAFP) screening.  **Exclusion criteria**: unable to be contacted, had incomplete follow-up information about pregnancy LTPA, delivered preterm, pre-pregnancy BMI was ≤ 18.5 kg/m2 or had unexplained high MSAFP levels at mid-pregnancy | **Early pregnancy weight**:  self-reported Pre-pregnancy weight self-report  **Late pregnancy weigh**t: Pre-birth weight (before delivery) extracted from medical records | Schlaff et al., 2014  [35] |
|  |  |  | **Inclusions criteria:** adult pregnant women who were receiving prenatal care at their respective locations of recruitment.  **Exclusion criteria**: women had pre-existing conditions or experienced complications during early pregnancy (e.g., habitual abortion, diabetes, high blood pressure, and hyperthyroidism) | **Early pregnancy weight**:  Pre-pregnancy extracted from medical records  **Late pregnancy weigh**t: Pre-delivery weight (before delivery) self-reported or extracted from medical records | Su et al., 2021  [41] |
|  |  | GWG was calculating the difference between pre-pregnancy weight during first trimester and the pre-birth weigh | **Inclusions criteria**: age≥ 20 years, singleton pregnancy  **Exclusion criteria**: gestational diabetes mellitus, hypertension heart disease, chronic renal disease, other diseases restricting physical activity. | **Early pregnancy weight**:  self-reported Pre-pregnancy weight  **Late pregnancy weight:**  measured Predelivery weight | Hong Jiang et al  2012  [27] |
|  |  | GWG calculated as by subtracting the antenatal weight form the pre-pregnancy weight in the ﬁrst prenatal visit before the 9 or the 11 weeks | **Inclusion criteria**: Singleton pregnancy, Prenatal care before the third trimester, age ≥18 years at the time of delivery, planned to deliver within the local hospital and keep the baby, Healthy and mentally competent. | **Early pregnancy weight:**  self-reported Pre-pregnancy weight  **Late pregnancy weight:** measured Predelivery weight | Olson et al., 2003  [21] |
|  |  | GWG were reported by women during the 1-month postpartum survey (how much weight they gained during pregnancy) | **Inclusions criteria**: singleton pregnancy, age 18-35 years, nulliparous.  **Exclusion criteria**: women who underweight (BMI <18.5 kg/m2 prior to pregnancy) and delivered prior to 37 weeks gestation, not provide information on height, pre-pregnancy weight or GWG. | Gestational weight gain:  Self-reported (baseline survey) | Kraschnewski et al.,  2013  [30] |
|  |  | GWG were estimated using the weight gained at the last day of weighing in pregnancy compared to the pre-pregnancy weight | **Exclusion criteria**: Not healthy, missing data for BMI for GWG | **Early pregnancy weight**:  self-reported Pre-pregnancy weight  **Late pregnancy weight**:  self-reported of weighting gained at last day of weighting | Merkx et al., 2015  [36] |
|  |  | GWG was then estimated by subtracting the pre-pregnancy weight from de last-measured weight before delivery in the last prenatal visit (38-40 weeks). | **Inclusions criteria**: age 18-35 years, in the third trimester of pregnancy, singleton pregnancy | **Early pregnancy weight:** measured and self-reported pre-pregnancy weight within 1-2 month(s) prior to pregnancy-booking  **Late pregnancy weight**: Measured pregnancy weight in the last prenatal visit (38-40 weeks). | Ebrahimi et al., 2015  [37] |
|  |  | GWG was defined as the difference between maternal weight at delivery and maternal pre-pregnancy weight up to 20 gestational weeks  at delivery | **Exclusion criteria**: current medications thought to adversely influence glucose tolerance, multiple gestation, history of diagnosis of diabetes, hypertension, heart disease or chronic renal disease, age <16 years or > 40 years | **Early pregnancy weight**:  self-reported Pre-pregnancy weight or extracted from medical records  **Late pregnancy weight:**  Pre-birth weight (before delivery) extracted from medical records | Chasan et al., 2014  [33] |
|  |  |  |  |  |  |
|  | Excessive GWG (>16 kg) | Weight gain was assessed electronically at the last clinic visit prior to delivery  (week 37) | **Inclusions criteria**: Before weeks 14-16 of gestation, Singleton pregnancy  **Exclusion criteria**: Pre-gestational diabetes, Congenital disorders of the babies, twin births, stillbirths and others adverse birth outcome. | **Early pregnancy weight**:  self-reported Pre-pregnancy weight  **Late pregnancy weight**:  Measured pregnancy weight in the last prenatal visit (37 w) | Haakstad et al., 2007  [22] |
|  | Excessive GWG (>15 kg) | GWG calculated by subtracting the pre-pregnancy weight from the predelivery weight | **Inclusions criteria:** age ≥18 years, singleton pregnancy | **Early pregnancy weight:**  Self-reported Pre-pregnancy weight  **Late pregnancy weight:**  Pre-birth weight (before delivery) extracted from medical records | Anh Vo Van Ha et al., 2020  [40] |
| Inadequate GWG | Inadequate GWG  below on the IOM guidelines 2009 | GWG calculated as by subtracting the antenatal weight form the pre-pregnancy weight in the ﬁrst prenatal visit before the 9 or the 11 (wk). | **Inclusion criteria**: Singleton pregnancy, Prenatal care before the third trimester, age ≥18 years at the time of delivery, planned to deliver within the local hospital and keep the baby, Healthy and mentally competent. | **Early pregnancy weight:**  self-reported Pre-pregnancy weight  **Late pregnancy weight:** measured Predelivery weight | Olson et al., 2003  [21] |
|  |  | GWG was calculated as the difference between weight at delivery and at the first antenatal clinic visit | **Inclusion criteria**: before 16 weeks gestation, from antenatal clinics and followed up until delivery  **Exclusion** criteria: age <15 years, preexisting diabetes mellitus and hypertension, multiple pregnancies. | **Early pregnancy weight**: Measured weight at the first antenatal clinic visit  **Late pregnancy weight:** Measured Weight at the at delivery | Abeysena et al., 2011  [26] |
|  |  | GWG was defined as pre-pregnancy weight was subtracted from weight at delivery | **Inclusions criteria**: age ≥ 15 years, with 6th–27th weeks of pregnancy, singleton pregnancy with no known chromosomal abnormality or birth defect, and no pre-pregnancy diabetes mellitus, maternal serum alpha-fetoprotein (MSAFP) screening.  **Exclusion criteria**: unable to be contacted, had incomplete follow-up information about pregnancy LTPA, delivered preterm, pre-pregnancy BMI was ≤ 18.5 kg/m2 or had unexplained high MSAFP levels at mid-pregnancy | **Early pregnancy weight**:  self-reported Pre-pregnancy weight self-report  **Late pregnancy weigh**t: Pre-birth weight (before delivery) extracted from medical records | Schlaff et al., 2014  [35] |
|  |  | GWG was defined as the difference between maternal weight at delivery and maternal pre-pregnancy weight up to 20 gestational weeks  at delivery total GWG | **Exclusion criteria**: current medications thought to adversely influence glucose tolerance, multiple gestation, history of diagnosis of diabetes, hypertension, heart disease or chronic renal disease, age <16 years or > 40 years | **Early pregnancy weight**:  self-reported Pre-pregnancy weight or extracted from medical records  **Late pregnancy weight:**  Pre-birth weight (before delivery) extracted from medical records | Chasan et al.,  2014  [33] |
|  |  | GWG were estimated using the weight gained at the last day of weighing in pregnancy compared to the pre-pregnancy weight | **Exclusion criteria**: Not healthy, missing data for BMI for GWG | **Early pregnancy weight**:  self-reported Pre-pregnancy weight  **Late pregnancy weight**:  self-reported of weighting gained at last day of weighting | Merkx et al., 2015  [36] |
|  |  | GWG was then estimated by subtracting the pre-pregnancy weight from de last-measured weight before delivery in the last prenatal visit (38-40 weeks). | **Inclusions criteria**: age 18-35 years, in the third trimester of pregnancy, singleton pregnancy | **Early pregnancy weight:** measured and self-reported pre-pregnancy weight within 1-2 month(s) prior to pregnancy-booking  **Late pregnancy weight**: Measured pregnancy weight in the last prenatal visit (38-40 weeks). | Ebrahimi et al., 2015  [37] |
|  | Different weight gain Groups | Weight gain was assessed electronically at the last clinic visit prior to delivery  (week 37) | **Inclusions criteria**: Before weeks 14-16 of gestation, Singleton pregnancy  **Exclusion criteria**: Pre-gestational diabetes, Congenital disorders of the babies, twin births, stillbirths and others adverse birth outcome. | **Early pregnancy weight**:  self-reported Pre-pregnancy weight  **Late pregnancy weight**:  Measured pregnancy weight in the last prenatal visit (37 w) | Haakstad et al., 2007  [22] |
| Rate of weight gain (kg/week) | **The continuous outcome variable Rate GWG** | | | | |
|  | Estimation of GWG per week | Average rate of weight gain per week (kg/week) | **Inclusions criteria**: their second and third trimesters without contraindications to exercise as per the PARMed-X for Pregnancy | **Early pregnancy weight**:  self-reported Pre-pregnancy weight  **Late pregnancy weight:** weight extracted from medical records (post-partum visit) | Cohen et al., 2013  [29] |
|  |  |  | **Inclusions criteria**: Pregnant women nulliparous pregnant women without BMI restrictions or BMI of >25 kg/m2 and singleton pregnancy*  **Exclusion criteria**: Recruitment after 20 weeks, age < 18 years, inadequate knowledge of the Dutch language, gestational diabetes mellitus before randomization, severe chronic disease | **Early pregnancy weight**:  self-reported Pre-pregnancy weight  **Late pregnancy weight:** measured Predelivery weight | Ruifrok et al., 2014  [32] |
|  |  | Rate of GWG was calculated as total pounds gained divided by gestational age at delivery. | **Exclusion criteria**: current medications thought to adversely influence glucose tolerance, multiple gestation, history of diagnosis of diabetes, hypertension, heart disease or chronic renal disease, age <16 years or > 40 years | **Early pregnancy weight**:  self-reported Pre-pregnancy weight or extracted from medical records  **Late pregnancy weight:**  Pre-birth weight (before delivery) extracted from medical records | Chasan et al., 2014  [33] |
|  | **Dichotomized outcome variable GWG** | | | | |
|  | Achieve appropriate weekly GWG | Weekly GWG was calculated using current pregnancy weight minus pre-pregnancy weight divided by gestational weeks minus twelve. | **Inclusion criteria**:>12 weeks gestation, free of medical risks for PA. | **Early pregnancy weight**:  Self-reported Pre-pregnancy weight  **Late pregnancy weight**: Current weight abstracted from medical records | Cohen et al.,  2009  [24] |
|  | Inadequate GWG rate  (below on the IOM guidelines 2009) | GWG rate in the second and third trimester was defined as the average weekly gain in that trimester | **Inclusions criteria**: age 20–40 years, gestation 13–36 weeks, singleton pregnancy, no medical conditions during pregnancy (e.g. gestational diabetes mellitus and pre-eclampsia) or chronic diseases (e.g. heart disease, renal disease and diabetes mellitus) | **Early pregnancy weight**:  Pre-pregnancy weight at second and third trimester weights extracted from medical records  **Late pregnancy weight**: Measured Current weight | Yong et al.,  2016  [38] |
|  | Excessive GWG rate  (exceeding IOM recommendations) | GWG for the second and third trimesters were calculated by subtracted weight at first visit from weight at 14–16 weeks of gestation and weight at the end of pregnancy and adjusted for the gestation at delivery (kg/week) | **Inclusions criteri**a: nulliparous women with singleton pregnancies at 14-16 weeks  **Exclusion criteria**: Miscarriage or termination 15–19 weeks, underweight prepregnancy (BMI<18.5 kg/m2), missing end of pregnancy weight, gestation for end pregnancy weight, anomalies or other reasons to termination >20 weeks | **Early pregnancy weight**: measured Pre-pregnancy weight  **Late pregnancy weight:** Measured pregnancy weight | Restall et al., 2014  [31] |
|  |  | GWG rate in the second and third trimester was defined as the average weekly gain in that trimester | **Inclusions criteria**: age 20–40 years, gestation 13–36 weeks, singleton pregnancy, no medical conditions during pregnancy (e.g. gestational diabetes mellitus and pre-eclampsia) or chronic diseases (e.g. heart disease, renal disease and diabetes mellitus) | **Early pregnancy weight**:  Pre-pregnancy weight at second and third trimester weights extracted from medical records  **Late pregnancy weight**: Measured Current weight | Yong et al.,  2016  [38] |
| GWG= gestational weight gain, BMI=body mass index, IOM guidelines= The Institute of Medicine guidelines  * The first cohort consisted of nulliparous pregnant women without BMI restrictions. The second cohort consisted of pregnant women with a BMI of >25 kg/m2 and at increased risk for GDM [32] | | | | | |
