# Supplementary material for: Physical activity and gestational weight gain: a systematic review of observational studies
Source: BMC Public Health. 2022 Oct 21;22:1951. doi: 10.1186/s12889-022-14324-0 (PMC9585865; doi:10.1186/s12889-022-14324-0)
Supplement: Supplementary file 2 — Supplementary Material 2 [file 12889_2022_14324_MOESM2_ESM.docx]

**Appendix 2 Table 3:** Physical activity dimensions assessed

We grouped the studies according Physical Activity Dimensions assessed: i) self-reported measure: duration, total physical activity, intensity, Level (ex. combination of duration +intensity), type of PA (Domains assessed: Walking, leisure time, occupational, transportation, and household), sedentary and others; ii) Objectively measure

| PA dimensions | Database PA | Dimensions assessed | PA variables | Assessment during pregnancy | Authors, year |  |  |
| --- | --- | --- | --- | --- | --- | --- | --- |
| Self-reported measure | | | | | | | |
| Duration | Interviewer administered Questionnaire  How many days per week and minutes per day they were engaging in regular physical activity since becoming pregnant and the type of PA | Engaging in regular physical activity since becoming pregnant | A 3-level variable  - inactive (< 60 min/ week)  - minimally active (60 – 149 min/week)  - active (150 min/week or more) | 1 period:  During the T3 (≥34 weeks gestation) | KRASCHNEWSKI et al 2013 [30] |  |  |
|  | Questionnaire modified from the Physical Activity Scale for the Elderly | Hours per week of total activity by summing walking, light-to-moderate, and vigorous activity. | Hours per week of total activity (min/d) | 1 period:  -At 26-28 weeks’ gestation (T2) during the previous 3 months | STUEBE A. et al 2009 [23] |  |  |
| Total PA | Validated, self-administered questionnaire: Pregnancy Physical Activity Questionnaire (PPAQ). | Total average MET-hr/wk was calculated using the sum of sedentary, light intensity, moderate-intensity, vigorous-intensity, household/caregiving, occupation and sports/exercise. | Meeting total PA guidelines:  Accumulated ≥8.5 MET-hr/wk | 3 periods:  T2, T3 and prenatal visit | Cohen T. et al  2009 [24] |  |  |
|  |  |  |  | 1 period:  27 ± 6 weeks gestation (One week) | MONPETIT et AL 2012 [28] |  |  |
|  |  |  |  | 1 period:  T2 (15- 26 Weeks) or T3 (27-38 weeks) | COHEN et al., 2013 [29] |  |  |
|  |  |  | Metting ACOG PA guidelines  Women with >7.5 MET hrs/week in any activity of moderate intensity or greater (30 min/d of activity at ≥ 3 METs multiplied by 5 d/W) | 3 periods:  - Early pregnancy (pre-pregnancy 1 year prior), Mid pregnancy (up to 20 gestational weeks), Late-pregnancy | CHASAN et al  2014 [33] |  |  |
|  |  |  | Total PA (MET-hr/day) divided into quartiles. | 3 periods  - Early pregnancy (pre-pregnancy 1 year prior), Mid pregnancy (up to 20 gestational weeks), Late-pregnancy | CHASAN et al  2014 [33] |  |  |
|  |  |  | Total PA (MET-hr/week) divided into tertiles | During pregnancy (NA) | ANH VO VAN HA et al 2020 [40] |  |  |
|  | Validated, self-administered questionnaire Global Physical Activity Questionnaire (GPAQ) | The total hours of PA were calculated and multiplied by MET hours per week. | Total average MET-hr/week categorized into 3 levels:  - Low PA,  - Moderate PA,  - high PA | 2 periods:  During Second (13–26 weeks) and third trimester (27–43 weeks) (last 7 days) | YONG et al  2016 [38] |  |  |
|  | Validated, self-administered questionnaire International Physical Activity Questionnaire (IPAQ) | Evaluated for each type of activity, the frequency and duration were assessed in days per week and minutes or hours per day respectively | Total MET (Min/week) | 1 period:  Last 7 days during T3 | EBRAHIMI et al., 2015 [37] |  |  |
| PA Intensity | Validated, self-administered questionnaire: Pregnancy Physical Activity Questionnaire (PPAQ). | Average metabolic equivalent (MET-hr/week) value was calculated using the duration of the time spent in each activity multiplied by the established categorical intensity value associated with the question. | Intensity score  Light, Moderate and vigorous  - Light intensity 1.5-3.0 MET-hr/week  - Moderate intensity 3.0-6.0 MET-hr/week  - Vigorous >6.0 MET-hr/week | 1 period:  T2 (15- 26 weeks) or T3 (27-38 weeks) | COHEN et al., 2013 [29] |  |  |
|  |  |  | Intensity score  Moderate and vigorous  - Moderate-intensity (3-6 METs) divided into quartile  - Vigorous intensity (>6 METs) score: dichotomized into as any vs none | 3 periods:  - Early pregnancy (pre-pregnancy 1 year prior), Mid pregnancy (up to 20 gestational weeks), Late-pregnancy | CHASAN et al  2014 [33] |  |  |
|  |  |  | Light and Moderate to vigorous  - Light (1.5 to <3 METs): divided into tertiles  - moderate-to-vigorous (≥3 METs) divided into tertiles | During pregnancy (NA) | ANH VO VAN HA et al  2020 [40] |  |  |
| PA level | Short questionnaire about PA of women during pregnancy compared PA before pregnancy. | Change in activity since pregnant according to the question:  How does the amount of PA you are getting now compare with your physical activity level before you got pregnant?” | 5 PA level:  - much less active  - little less active  - Same  - little more active  - much more active | During pregnancy (NA) | Olson et al., 2003  [21] |  |  |
|  | Questionnaire modified from the Physical Activity Scale for the Elderly | Duration of moderate activity and vigorous activity | 2 PA level  Moderate activity (Min/day)  Vigorous activity (Min/day) | 1 period:  At 26-28 weeks gestation (T2) during the previous 3 months | STUEBE A. et al 2009  [23] |  |  |
|  | General Practice Physical  Activity Questionnaire (GPPAQ) | Duration of moderate activity | 4 PA levels:  - Inactive  - Somewhat active  - Moderately active  - Active (150 min/week)- achieving the recommended | 1 period:  Last week during mid-pregnancy  (26-28 weeks) | Collings et al  2020  [39] |  |  |
|  | Short questionnaire about PA level of pregnant women | Vigorous recreational PA  PA level evaluated by combining duration, intensity and frequency:  How long they had been  exercising regularly and their 3 most preferred activities  Duration, frequency and intensity of the training sessions? | 3 PA level  - Low level (no sweating and normal breathing)  - Moderate level (modestly sweating and light breathing)  - High level (sweating and breathing heavily) | 3 periods:  T1, T2, T3 retrospectively | HAAKSTAD et al 2007  [22] |  |  |
|  | LTPA questionnaire | LTPA level evaluated by combining duration, intensity.  Days per week and typical time (hours and minutes) spent doing moderate and vigorous activities in one day. Minutes per day of moderate and vigorous LTPA were each multiplied by the number of days per week to obtain minutes per week of moderate and vigorous LTPA. | Dichotomised variable of LTPA (Min/week)  - moderate LTPA: active vs not active  - vigorous LTPA: active vs not active | 1 period:  At the time of study enrolment (prior to 14 week) | SCHLAFF et al  MARS 2014  [34] |  |  |
|  | Validated, self-administered questionnaire International Physical Activity Questionnaire- Short Form (IPAQ-SF) | Average metabolic equivalent (MET-hr/week) value was calculated using the duration of the time spent in each activity multiplied by the established categorical intensity value associated with the question. | Decline in PA during pregnancy (METs-min/week):  - No decline  - 1–600  - 601–4000  - >4000 | 1 period:  During second and third trimesters of pregnancy | Sun et al., 2021  [41] |  |  |
| Type of PA | Short questionnaire-  How long they had been exercising regularly and their 3 most preferred activities, Duration, frequency and intensity of the training sessions | Duration of recreational Exercise | - Exercise duration (‹30min,30-60, ›60min) | 3 periods:  T1, T2, T3 retrospectively | HAAKSTAD et al 2007  [22] |  |  |
|  | Questionnaire modified from the Physical Activity Scale for the Elderly | Duration of walking | walking (30 Min/day) | 1 period:  At 26-28 weeks gestation (T2) during the previous 3 months | STUEBE A. et al 2009  [23] |  |  |
|  | Short, self-administered questionnaire | LTPA: MET intensities (Kcal/kg/hr)  PA performed most often during a typical week in their leisure time and report type, average duration, and average frequency of up to two activities | - Meeting recommendations during pregnancy (≥7.5 kcal/kg/week recommended”). | 1 period:  typical week in their leisure time | SCHLAFF et al  NOVEMBRE 2014  [35] |  |  |
|  | Interviewer-administered questionnaire. | Duration of different postures adopted in terms of walking, sitting standing and sleeping per day. | 3 variables:  - Standing > 2.5 h/day  - Walking > 2.5 h/day  - Standing and walking ≥5 h/day | 3 periods:  T1, T2, T3 retrospectively (12, 28 and 36 weeks gestation) | ABEYSENA et al  2011  [26] |  |  |
|  | Validated, self-administered questionnaire: Pregnancy Physical Activity Questionnaire (PPAQ). | Average metabolic equivalent (MET-hr/week) value was calculated using the duration of the time spent in each activity multiplied by the established categorical intensity value associated with the question. | 3 domains: (MET-hrs/week)  - household/ caregiving,  - occupation  - sports/exercise | 1 period:  - T2 (15- 26 weeks) or T3 (27-38 weeks) | COHEN et al., 2013  [29] |  |  |
|  |  |  | 3 domains: (MET-hrs/day)  - household/caregiving: divided into quartile  - sport-exercise: divided into tertile  - occupation: divided into 3 categories according to median MET-hrs/day. | 3 periods:  - Early pregnancy (pre-pregnancy 1 year prior), Mid pregnancy (up to 20 gestational weeks), Late-pregnancy | CHASAN et al  2014  [33] |  |  |
|  |  |  | 3 domains: (MET-hrs/week)  - household/caregiving: divided into tertiles  - Occupational: divided into tertiles  - Transportation: divided into tertiles | During pregnancy (NA) | ANH VO VAN HA et al  2020  [40] |  |  |
| Sedentary | Short, self-administered questionnaire | PA performed most often during a typical week in their leisure time and report type, average duration, and average frequency of up to two activities | Sedentary score:  Insufficiently active (<7.5 kcal/kg/week, “low”) | 1 period:  Typical week in their leisure time while pregnant | SCHLAFF et al  NOVEMBRE 2014  [35] |  |  |
|  | Questionnaire modified from the Physical Activity Scale for the Elderly | We defined sedentary lifestyle according to the duration /week of day. | 2 Sedentary score:  - sedentary lifestyle as < 2.5 hours per week, or<22 minutes per day, of total activity.  - TV watching hours per week | 1 period:  At 26-28 weeks gestation (T2) during the previous 3 months | STUEBE A. et al 2009  [23] |  |  |
|  | Validated, self-administered questionnaire: Pregnancy Physical Activity Questionnaire (PPAQ). | Average metabolic equivalent (MET-hr/week) value was calculated using the duration of the time spent in each activity multiplied by the established categorical intensity value associated with the question. | Sedentary behaviors <1.5 METs | 1 period:  - T2 (15- 26 weeks) or T3 (27-38 weeks) | COHEN et al., 2013 [29] |  |  |
|  |  |  | Sedentary <1.5 METs: divided into quartiles | 3 periods:  - Early pregnancy, (pre-pregnancy 1 year prior), - Mid pregnancy (up to 20 gestational weeks), Late-pregnancy | CHASAN et al  2014 [33] |  |  |
|  | Short, self-administered questionnaire | Evaluation of the time spend performing sedentary activities during the last three months of the pregnancy | Total sitting time (hours/week): divided into tertile | During pregnancy (NA) | ANH VO VAN HA et al  2020 [40] |  |  |
|  | Validated, self-administered questionnaire International Physical Activity Questionnaire (IPAQ) | Evaluation of the time spend sitting in days. | Sitting (min/days) | 1 period:  Last 7 days during T3 | EBRAHIMI et al., 2015  [37] |  |  |
| Others dimensions | Interviewer administered questionnaire. | Evaluation of decline PA during pregnancy | Exercise in pregnancy:  - Unchanged PA  - Increased PA  - Decreased PA | 1 period:  14–16 weeks of gestation | RESTALL et al  2014  [31] |  |  |
|  | Short, self-administered questionnaire | Evaluation of whether or not they reduced their PA during pregnancy. | Decline in PA (yes vs no) | During pregnancy (NA) | MERKX et al  2015 [36] |  |  |
|  | Short, self-administered questionnaire | - to rate their pre-pregnancy activity | 2 variables  - Pre-pregnancy PA (1= not active at 7= very active) | During pregnancy (NA) | MERKX et al  2015 [36] |  |  |
|  | No standard questionnaire | Evaluation of the motivation of pregnant women. By  -how many minutes in last week walked, engaged sport, undertook short rigorous activities or work in the home and garden | - Motivation healthy PA “having at least five time a week 30 min a day PA which makes your breath more intense.”  (1= totally disagree to 7= totally agree) | During pregnancy (NA) | MERKX et al  2015 [36] |  |  |
| Objective PA measure | | | | | | |  |
| Daily steps | Pedometer logbook (The New Lifestyles Digi-Walker SW 200) record daily steps. | Counts of steps per day | Daily pedometer step counts (per 1000 steps per day) | 1 period:  One week  27 ± 6 weeks gestation | MONPETIT et AL. 2012  [28] |  |  |
|  | Pedometer Omron HJ-005 measured step counts/day |  | Daily step divided into 4 categories.  Active level according to daily step counts  - Sedentary (< 5000 daily steps)  - Low Active (5000~7500 daily steps)  - Somewhat Active (7500~10000 daily steps)  - Active (≥ 10000 daily steps) | 2 period:  -T2:18-28 weeks, T3: 29-35 weeks (for 4 days: 2 working days and 2 weekend days) | HONG JIANG et al 2012  [27] |  |  |
|  | Pedometer New Lifestyles Digi-Walker SW-200 |  | Accumulated >=7500 steps/day | 3 period:  One week per T2, T3 and prenatal visit | Cohen T. et al 2009 [24] |  |  |
| PA level | Accelerometer the Actiheart detected movement was expressed as the mean count of vertical accelerations per Minutes. | time spent in moderate PA: (any activity between 3-6 METs). | 2 groupes PA Based on the ACOG recommendations:  - active ≥30 min of moderate PA/day  - inactive <30 min of moderate PA/day | 1 period:  35-41 weeks of gestation, 5 consecutive days | Melzer K, et al  2010  [25] |  |  |
|  | PA measured using accelerometer (ActiTrainer accelerometer ActiGraph) | time spent in moderate-to-vigorous physical activity (MVPA= 2020 to 5998 counts/min) | % of time spent in MVPA (Moderate to vigorous PA) = 2020 to 5998 counts/min) | 2 periods during pregnancy:  At 15 weeks, to 32–35 weeks | RUIFROK et al., 2014  [32] |  |  |
| Sedentary behaviors | Pedometer Omron HJ-005 measured step counts/day | Counts of steps per day | - Sedentary (< 5000 daily steps) | 2 period:  -T2:18-28 weeks, T3: 29-35 weeks, (for 4 days: 2 working days and 2 weekend days) | HONG JIANG et al 2012  [27] |  |  |
|  | PA measured using accelerometer.  (ActiTrainer accelerometer ActiGraph) | time spent in sedentary time (<100 counts/min). | % of time spent in Sedentary behavior <100 counts/min | 2 periods during pregnancy:  At 15 weeks, to 32–35 weeks | RUIFROK et al., 2014  [32] |  |  |

hr=hours; METs = Metabolic Equivalent of Task
