# Supplementary material for: Physical activity and gestational weight gain: a systematic review of observational studies
Source: BMC Public Health. 2022 Oct 21;22:1951. doi: 10.1186/s12889-022-14324-0 (PMC9585865; doi:10.1186/s12889-022-14324-0)
Supplement: Supplementary file 3 — Supplementary Material 3 [file 12889_2022_14324_MOESM3_ESM.docx]

**Appendix 3 Table 4 Overview of current evidence concerning possible effects on GWG of PA**

| Type(s) or subtype(s) | PA dimension | PA variables | Statistical model | Results | Authors, date |
| --- | --- | --- | --- | --- | --- |
| GWG as continuous variable | | | | | |
| Total gestational weight gain | **Objectif PA measure** | PA level according daily step counts  Low Active (5000~7500 daily steps),  Somewhat Active (7500~10000 daily steps)  Active (≥ 10000 daily steps) | Multiple linear regression | ***PA during Trimester 2***  Low active: Beta= -0.02 [-0.88; 0.83]  Somewhat active: **Beta= -0.85**[ **-1.69; 0.00**]  **Active: Beta= -1.41[ -2.37; -0.45]**  ***PA during Trimester 3***  Low active  Beta= -0.52[-1.24 ; 0.20]  **Somewhat active: Beta= -0.81**[**-1.62 ; 0.01**]  **Active: Beta= -1.62**[ **-2.66 ; -0.57**]  ***PA during the last 2 Trimester***  Low active: Beta= 0.18 [ -0.60; 0.96]  Somewhat active: Beta= -0.47 [-1.29; 0.36]  **Active: Beta= -1.45** [**-2.44; -0.46**] | HONG JIANG et al  2012  (28) |
|  |  | PA intensity:  MVPA (Moderate to vigorous PA) | Regression models | %MVPA 15 weeks of gestation  Beta=−0.07 [−0.48; 0.34]  Change from 15 to 32–35 weeks of gestation  % MVPA  Beta −0.16 [−0.47;0.15] | RUIFROK ET AL., 2014  (33) |
|  |  | Sedentary behaviour <100 counts/min | Regression models | 15 weeks of gestation  Beta= −0.07 [−0.15; 0.01]  Change from 15 to 32–35 weeks of gestation between % sedentary behaviour  Beta −0.02 [−0.12; 0.07] | RUIFROK ET AL., 2014  (33) |
|  |  | Daily pedometer step counts (per 1000 steps per day) | Hierarchical  multiple regression analyses | Beta=-0.60 p=0.107 (per 1000 steps/d) | MONPETIT et AL  2012 (29) |
|  | Self-reported PA | Total PA MET-hr/wk (by tertile) | multiple linear regression models | Tertile 1: Ref  **Tertile 2: Beta= - 0.59 [-1.06; -0.12]**  **Tertile 3: Beta= - 0.53 [ -1.03; -0.04]** | ANH VO VAN HA ET AL  2020 (41) |
|  |  | PA Intensity (MET-hours/week)  - Light (1.5 to <3 METs) (by tertile)  - moderate-to-vigorous (≥3 METs) (by tertile) | multiple linear regression models | ***Light intensity***  Tertile 1: Ref  Tertile 2: beta= -0.27 [-0.73; 0.18]  Tertile 3: beta= -0.44 [-0.90; 0.02]  ***Moderate-to-vigorous***  Tertile 1: Ref  Tertile 2: beta= -0.32 [-0.77; 0.13]  **Tertile 3: beta= -0.56 [-1.01; -0.11]** | ANH VO VAN HA ET AL  2020  (41) |
|  |  | Type of PA (MET-hours/week)  - household/caregiving (by tertile)  - Occupational (by tertile)  - Transportation (by tertile) | multiple linear regression models | **Household/caregiving** Tertile 1: Ref  Tertile 2: beta= -0.37 [ -0.82; 0.08]  **Tertile 3: beta= -0.63** [ **-1.11; -0.16**]  **Occupational:** Tertile 1: Ref  Tertile 2: beta=-0.35 [ -0.89; 0.18]  **Tertile 3**: **beta=-0.79** [**-1.35; -0.23**]  **Transportation** Tertile 1: Ref  Tertile 2: beta=0.27 [-0.17; 0.72]  Tertile 3 : beta= 0.09 [ -0.36; 0.55] | ANH VO VAN HA ET AL  2020  (41) |
|  |  | Sedentary behaviors:  Sitting time (hs/w) (by tertile) | Multiple linear regression models | Sitting time (hs/w): Tertile 1: Ref  Tertile 2: beta=-0.05 [ -0.50; 0.41]  **Tertile 3 : beta= 0.62** [**0.12; 1.12**] | ANH VO VAN HA ET AL 2020  (41) |
|  |  | PA duration (30min/d) | Multivariable logistic regression | OR= -0.48; [ -1.01 to 0.04] kg per 30 minutes per day) | STUEBE A. ET AL 2009 (24) |
|  |  | PA Level | Multivariable logistic regression | **Duration of vigorous activity**  Beta= -0.37 [-0.90; 0.17]  **Duration of moderate activity**  OR= -0.12 [-0.27; - 0.002] | STUEBE A. ET AL 2009  (24) |
|  |  | Type PA: Walking (30 min/d) | Multivariable logistic regression | Walking (30 min/d)  **Beta= -0.20 [-0.43; 0.04]** | STUEBE A. ET AL 2009 (24) |
|  |  | Total Met-hr/day (by quartile) |  | ***Total PA during Early Pregnancy***  **Quartile 2 Beta = 3.35 p= 0.05**  **Quartile 3 Beta = 3.57 p= 0.03**  Quartile 4 Beta =2.36 p= 0.17  ***Total PA during mid pregnancy***  Quartile 2 Beta = 2.82 p= 0.12  Quartile 3 Beta = 3.00 p= 0.10  Quartile 4 Beta = 2.03 p= 0.27  ***Total PA during Late pregnancy***  Quartile 2 Beta = -1.80 p= 0.30  Quartile 3 Beta = -2.32 p= 0.18  Quartile 4 Beta = -1.76 p= 0.32 | CHASAN ET AL  2014  (34) |
|  |  | Total PA: Met PA guideline (>7.5 MET hrs/week) | Multinomial logistic regression  Linear regression models | ***Early Pregnancy***  NO Met Physical Activity Guidelines Met PA guidelines: Beta= -1.33 p=0.36  ***Mid pregnancy***  NO Met Physical Activity Guidelines Met PA guidelines: Beta = 0.36 p= 0.82  ***Late pregnancy***  **NO Met Physical Activity Guidelines Met PA guidelines: Beta = 3.62 p=0.01** | CHASAN ET AL  2014  (34) |
| Dichotomized outcome variable GWG | | | | | |
| Excessive GWG | Objective PA Measure | PA level according daily step counts  Low Active (5000~7500 daily steps),  Somewhat Active (7500~10000 daily steps)  Active (≥ 10000 daily steps) | Multiple binary logistic | ***PA during Trimester 2-*** Sedentary: Ref  Low active OR= 0.95 [ 0.62; 1.46]  Somewhat active OR= 0.77 [ 0.50; 1.17]  **Active OR= 0.59** [ **0.36;0.95**]  ***PA during Trimester 3-*** Sedentary: Ref  Low active OR= 0.72 [ 0.50; 1.05]  Somewhat active OR= 0.66 [ 0.43; 1.00]  Active OR= 0.62 [ 0.36; 1.06]  ***PA during the last 2 Trimester*** Sedentary: Ref  Low active OR= 1.05 [ 0.68; 1.62]  Somewhat active OR= 0.85 [ 0.54; 1.34]  Active OR= 0.60 [ 0.35; 1.03] | HONG JIANG et al  2012  (28) |
|  | **Self-reported PA measure** | Total PA (30 min/d) | Multivariable logistic regression | OR=0.95 [ 0.89;1.01] | STUEBE A. ET AL 2009 (24) |
|  |  | Level PA (30 min/d) | Multivariable logistic regression | **Vigorous activity OR= 0.76** [**0.60;0.96**]  Moderate activity OR= 1.00 [ 0.85;1.17] | STUEBE A. ET AL 2009 (24) |
|  |  | Type PA (30 min/d): | Multivariable logistic regression | Walking OR= 0.92 [0.83; 1.01]  TV watching OR=0.98 [0.93;1.02] | STUEBE A. ET AL 2009 (24) |
|  |  | Sedentary lifestyle as < 2.5 hours per week, or<22 minutes per day, of total activity | Multivariable logistic regression | OR= 1.26 [0.95;1.69] | STUEBE A. ET AL 2009 (24) |
|  |  | Level LTPA (Min/w) | Logistic regression model | *Moderate LTPA (ref: not active)*  Active  OR=1.22 [ 0.58; 2.58] - ARCH GWG  OR=1.23 [0.59; 2.57]- birth certificate  *Vigorous LTPA (ref: not active)*  Active  OR=1.10 [ 0.41; 2.91] - ARCH GWG  OR= 1.33 [ 0.51; 3.51]- birth certificate | SCHLAFF ET AL  MARS 2014  (35) |
|  |  | MET intensities (Kcal/kg/hr) divided into 3 categories  - none LTPA: Sedentary  - Low LTPA: (<7.5 kcal/kg/wk): insufficiently active  - recommended LTPA (≥7.5 kcal/kg/wk | Logistic regression | Low LTPA  OR= 0.65 [0.37; 1.14]  Recommended LTPA  OR= 0.84 [0.49; 1.43] | SCHLAFF et al  NOVEMBRE 2014  (36) |
|  |  | Level PA  - much less active  - little less active  - Same pa  - little more active  - much more active | Multiple linear and logistic regression model | Less physical activity  Excessive GWG vs in range  **OR=1.68** [**1.1; 2.6**] | OLSON ET AL., 2003  (22) |
|  |  | Total PA  inactive (< 60 min/ week)  minimally active (60 – 149 min/week)  active (150 min/week or more) | Multivariable logistic regression | Minimally active OR= 0.97 [ 0.79;1.2]  **Active OR= 0.71**[ **0.57; 0.88**]  Inactive (>60min/wk): REF | KRASCHNEWSKI ET AL 2013  (31) |
|  |  | Exercise in pregnancy:  - Unchanged PA  - Increased PA  - Decreased PA | Logistic regression | Unchanged: Ref  Increased OR= 0.91 [ 0.55;1.51]  **Decreased OR= 1.30** [ **1.01;1.69**] | RESTALL ET AL  2014  (32) |
|  |  | -Total Met-hr/day | Multinomial logistic regression  Linear regression model | ***Total PA during Early Pregnancy***  - Quartile 2 OR = 1.04 [ 0.64; 1.68]  - Quartile 3 OR = 1.13 [ 0.69; 1.83]  - Quartile 4 OR = 1.24 [ 0.74; 2.06]  ***Total PA during mid pregnancy***  **- Quartile 2 OR= 1.92** [**1.16; 3.16**]  **- Quartile 3 OR= 1.97** [**1.19; 3.27**]  - Quartile 4 OR = 1.22 [0.73; 2.02]  ***Total PA during Late pregnancy***  - Quartile 2 OR = 0.87 [0.52; 1.46]  - Quartile 3 OR= 0.85 [0.51; 1.42]  - Quartile 4 OR = 0.73 [ 0.44; 1.22] | CHASAN ET AL  2014  (34) |
|  |  | Met PA guideline (>7.5 MET hrs/week) | Multinomial logistic regression  Linear regression models | ***Early Pregnancy***  NO Met Physical Activity Guidelines Met PA guidelines OR= 1.17 [0.76; 1.79]  ***Mid pregnancy***  NO Met Physical Activity Guidelines Met PA guidelines OR= 1.15 [0.73; 1.82]  ***Late pregnancy***  NO Met Physical Activity Guidelines Met PA guidelines OR = 1.29 [0.83; 2.00] | CHASAN ET AL  2014  (34) |
|  |  | Sedentary <1.5 METs- | Multinomial logistic regression  Linear regression models | ***Early Pregnancy***  Quartile 2 OR = 1.34 [0.83; 2.18]  Quartile 3 OR = 1.26 [0.79; 2.01]  Quartile 4 OR = 1.06 [0.66; 1.70]  ***Mid pregnancy***  Quartile 2 OR = 1.11[0.68; 1.80]  Quartile 3 OR = 1.44 [ 0.88; 2.37]  Quartile 4 OR = 1.05 [0.64; 1.72]  ***Late pregnancy***  Quartile 2 OR = 0.92 [0.56; 1.53]  Quartile 3 OR = 0.93 [0.56; 1.50]  Quartile 4 OR= 0.91 [0.55; 1.51] | CHASAN ET AL  2014  (34) |
|  |  | PA intensity (by quartile)  Moderate-intensity 3-6 METs  Vigorous intensity >6 METs | Multinomial logistic regression  Linear regression models | ***Early Pregnancy***  Moderate  Quartile 2 OR = 1.08 [0.67; 1.76]  Quartile 3 OR = 0.76 [ 0.47; 1.23]  Quartile 4 OR = 1.26 [0.76; 2.10]  Vigorous- Any: OR = 0.91 [ 0.55; 1.50]  ***Mid pregnancy***  Moderate  Quartile 2 OR = 1.16 [0.70; 1.91]  Quartile 3 OR = 1.54 [ 0.92; 2.58]  Quartile 4 OR = 0.95 [ 0.57; 1.57]  Vigorous- Any: OR = 0.89 [0.52; 1.52]  ***Late pregnancy***  Moderate  Quartile 2 OR = 0.87 [0.52; 1.44]  Quartile 3 OR = 0.65 [0.40; 1.07]  Quartile 4 OR= 0.81 [0.49; 1.34]  Vigorous: Any*:* OR = 0.99 [0.58; 1.71] | CHASAN ET AL  2014  (34) |
|  |  | Type PA | Multinomial logistic regression  Linear regression models | ***Early Pregnancy***  - Sports/Exercise  Low OR = 0.76 [0.49; 1.18]  High OR = 0.84 [0.57; 1.23]  - Household/Caregiving  Quartile 2 OR = 1.07 [ 0.66; 1.75]  Quartile 3 OR = 1.03 [0.61; 1.72]  Quartile 4 OR = 0.97 [ 0.57; 1.67]  - Occupation  Low OR = 0.97 [0.57; 1.67]  High OR = 1.21 [ 0.83; 1.77]  ***Mid pregnancy***  - Sports/Exercise  Low OR = 1.34 [ 0.88; 2.06]  High OR = 1.34 [0.89; 2.02]  - Household/Caregiving  Quartile 2 OR = 0.83 [ 0.50; 1.37]  Quartile 3 OR = 1.44 [0.85; 2.43]  Quartile 4 OR = 0.95 [0.55; 1.64]  - Occupation  Low OR = 0.96 [ 0.57; 1.61]  High OR = 1.00 [0.68; 1.47]  ***Late pregnancy***  - Sports/Exercise  Low OR = 0.82 [0.53; 1.26]  High OR = 0.81 [0.54; 1.24]  - Household/Caregiving  Quartile 2 OR = 1.00 [ 0.60; 1.66]  Quartile 3 OR = 0.91 [0.54; 1.53]  Quartile 4 OR = 0.77 [0.45; 1.32]  - Occupation  Low OR = 0.71 [0.37;1.37]  High OR = 0.73 [0.50; 1.06] | CHASAN ET AL  2014  (34) |
|  |  | Level of motivation healthy PA:  - Rate pre-pregnancy activity: not active vs active  - Reduce vs not PA during pregnancy | Multinomial logistic regression | Motivation healthy PA OR=0.90 [0.73;1.11]  **Decline in PA OR=0.54** [ **0.33;0.89**] | MERKX ET AL  2015  (37) |
|  |  | Decline in PA during pregnancy (METs-min/week):  - No decline  - 1–600  - 601–4000  - >4000 | Multivariate logistic regression | Decline in PA  No decline: Ref 1–600 OR=1.18 [0.77–1.80] 601–4000 OR=1.13 [0.77–1.66] **>4000 OR= 2.38 [1.27–4.43]** | SUN et al., 2021  [41] |
| Execessive GWG (>15 kg) |  | Sedentarity | Multiple linear regression models | Spending more time sitting during pregnancy could increase the risk of the excessive GWG **OR=1.73,** [ **1.27; 2.36**] | ANH VO VAN HA ET AL 2020  (41) |
| Inadequate GWG | Self’reported PA | Type of PA  - Standing > 2.5 h/day  - Walking > 2.5 h/day  - Sitting >3.5 h/day  - Standing and walking ≥5 h/day | Multivariate logistic regression | ***Trimester 1:***  **Standing OR= 1.53 [1.09; 2.15]**  Walking OR= 1.34 [ 0.97; 1.85]  **Sitting OR= 1.64 [1.19; 2.25]**  **Standing and walking OR= 1.53 [1.13; 2.07]**  ***Trimester 2:***  Standing OR= 1.27 [0.87; 1.84]  **Walking OR= 1.51 [1.07; 2.14]**  Sitting OR= 1.30 [0.94; 1.80]  **Standing and walking OR= 1.50 [1.08; 2.07]**  ***Trimester*** 3  Standing OR= 1.26 [0.84; 1.89]  Walking OR= 1.21 [0.85; 1.71]  Sitting OR= 1.13 [0.81; 1.58]  Standing and walking OR= 1.19 [0.85; 1.66]  **Standing and walking during the Trimester 1 and Trimester 2**  **OR= 1.50 [ 1.04; 2.15]** | ABEYSENA ET AL  2011  (27) |
|  |  | Met PA guideline (>7.5 MET Hrs/week) | Multinomial logistic regression  Linear regression models | ***Early Pregnancy***  NO Met Physical Activity Guidelines Met PA guidelines OR= 1.34 [0.82; 2.22]  ***Mid pregnancy***  NO Met Physical Activity Guidelines Met PA guidelines OR= 1.11 [ 0.65; 1.92]  ***Late pregnancy***  NO Met Physical Activity Guidelines Met PA guidelines OR = 0.95 [0.53; 1.71] | CHASAN ET AL  2014  (34) |
|  |  | Total Met-hr/day | Multinomial logistic regression  Linear regression models | ***Total PA during Early Pregnancy***  Quartile 2 OR = 0.63 [0.36; 1.11]  Quartile 3 OR = 0.65 [0.37; 1.15]  Quartile 4 OR = 0.98 [0.55; 1.73]  ***PA during Mid pregnancy***  Quartile 2 OR= 1.13 [ 0.62; 2.04]  Quartile 3 OR= 1.13 [ 0.63; 2.05]  Quartile 4 OR = 1.06 [0.60; 1.80]  ***PA during the late pregnancy***  Quartile 2 OR = 0.91 [ 0.48; 1.73]  Quartile 3 OR= 0.86 [ 0.45; 1.64]  Quartile 4 OR = 0.73 [0.38; 1.40] | CHASAN ET AL  2014  (34) |
|  |  | Sedentary <1.5 METs- | Multinomial logistic regression  Linear regression models | ***Early Pregnancy***  Quartile 2 OR = 1.12 [ 0.64; 1.95]  Quartile 3 OR = 0.93 [ 0.54; 1.62]  Quartile 4 OR = 0.83 [ 0.48; 1.45]  ***Mid pregnancy***  Quartile 2 OR = 1.06 [ 0.59; 1.89]  Quartile 3 OR = 1.39 [ 0.77; 2.49]  Quartile 4 OR =1.27 [ 0.72; 2.26]  ***Late pregnancy***  Quartile 2 OR = 0.55 [0.30; 1.03]  Quartile 3 OR = 0.55 [ 0.29; 1.03]  Quartile 4 OR= 0.64 [ 0.35; 1.18] | CHASAN ET AL  2014  (34) |
|  |  | PA intensity (by quartile)  Moderate-intensity 3-6 METs  Vigorous intensity >6 METs | Multinomial logistic regression  Linear regression models | ***Early Pregnancy***  Moderate  Quartile 2 OR = 0.84 [0.47; 1.48]  Quartile 3 OR =0.67 [0.38; 1.17]  Quartile 4 OR = 1.03 [ 0.57; 1.86]  Vigorous: ANY OR = 0.76 [0.41. 1.41]  ***Mid pregnancy***  Moderate  Quartile 2 OR = 0.78 [0.43.; 1.39]  Quartile 3 OR = 0.78 [ 0.42; 1.43]  Quartile 4 OR = 0.79 [ 0.45; 1.41]  Vigorous: ANY OR = 1.02 [ 0.55; 1.88]  ***Late pregnancy***  Moderate  Quartile 2 OR =1.01 [0.54; 1.89]  Quartile 3 OR = 0.59 [0.31; 1.12]  Quartile 4 OR=0.86 [0.46; 1.61]  Vigorous: ANY OR = 0.93 [0.47; 1.85] | CHASAN ET AL  2014  (34) |
|  |  | Type PA | Multinomial logistic regression  Linear regression models | ***Early Pregnancy***  - Sports/Exercise  Low OR = 0.80 [0.47; 1.34]  High OR =0.83 [0.53; 1.30]  ***- Household/Caregiving***  **Quartile 2 OR = 0.49** [**0.27; 0.89**]  Quartile 3 OR = 0.75 [ 0.41; 1.36]  Quartile 4 OR = 0.90 [0.49; 1.67]  - Occupation  Low OR = 0.87 [0.51; 1.46]  High OR = 0.87 [0.55; 1.38]  ***Mid pregnancy***  - Sports/Exercise  Low OR = 0.88 [0.53; 1.47]  High OR = 1.08 [0.67; 1.75]  - Household/Caregiving  Quartile 2 OR = 0.67 [0.37; 1.20]  Quartile 3 OR = 0.63 [0.33; 1.20]  Quartile 4 OR = 0.95 [0.51; 1.76]  - Occupation  Low OR = 0.89 [0.48; 1.66]  High OR = 0.94 [0.60; 1.49]  ***Late pregnancy***  - Sports/Exercise  Low OR = 0.70 [0.40; 1.21]  High OR = 0.80 [0.48; 1.34]  - Household/Caregiving  Quartile 2 OR = 1.03 [0.54; 1.97]  Quartile 3 OR = 0.93 [0.48; 1.83]  Quartile 4 OR = 1.06 [ 0.54; 2.09]  - Occupation  Low OR = 1.39 [0.68; 2.84]  High OR = 0.50 [0.30; 0.84] | CHASAN ET AL  2014  (34) |
|  |  | - Level of motivation healthy PA  - Reduce vs not PA during pregnancy | Multinomial logistic regression | Motivation healthy PA  OR= 0.98 [ 0.73; 1.31]  Decline in PA  OR= 0.80 [0.41; 1.59] | MERKX ET AL  2015  (37) |
|  |  | Total PA  Total MET-Min/w | Logistic regression models | OR=1.001 [1.000;1.002] | EBRAHIMI ET AL., 2015 (38) |
|  |  | Sedentary  Sitting (min/days) | Logistic regression models | **OR=0.997** [ **0.994; -0.999**] | EBRAHIMI ET AL., 2015 (38) |
| GWG rate | | | | | |
| GWG rate (continuous variable) | Objective PA measure | Sedentary behavior  ( <100 counts/min) | Regression models | 15 weeks of gestation  Beta −0.004 [−0.01; 0.001]  Change from 15 to 32–35 weeks of gestation between % sedentary behaviour  Beta= −0.001 [−0.01; 0.004] | RUIFROK ET AL., 2014 (33) |
|  | Self’reported PA | PA intensity:  MVPA (Moderate to vigorous PA) | Regression models | 15 weeks of gestation  Beta= −0.002 [−0.02; 0.02]  Change from 15 to 32–35 weeks of gestation between % MVPA  Beta −0.01 [ −0.03; 0.01] | RUIFROK ET AL., 2014 (33) |
|  |  | Met PA guideline (>7.5 MET hrs/week)  -Total Met-hr/day  PA by intensity (by quartile)  -Sedentary <1.5 METs-Moderate-intensity 3-6 METs  -Vigorous intensity >6 METsPA by type (by quartile) household/caregiving activities, sport-related activities occupationally related activities, | Multinomial logistic regression  Linear regression models | ***Early Pregnancy***  NO Met Physical Activity Guidelines Met PA guidelines Beta= -0.03 p= 0.47  ***Mid pregnancy***  NO Met Physical Activity Guidelines Met PA guidelines Beta = 0.01 p=0.72  ***Late pregnancy***  NO Met Physical Activity Guidelines Met PA guidelines **Beta = 0.08 p= 0.03** | CHASAN ET AL  2014  (34) |
|  |  | Met PA guideline (>7.5 MET hrs/week)  -Total Met-hr/day  PA by intensity (by quartile)  -Sedentary <1.5 METs-Moderate-intensity 3-6 METs  -Vigorous intensity >6 METsPA by type (by quartile) household/caregiving activities, sport-related activities occupationally related activities, | Multinomial logistic regression  Linear regression models | ***Total PA during early Pregnancy***  **Quartile 2 Beta = 0.08 p= 0.05**  **Quartile 3 Beta = 0.08 p= 0.05**  Quartile 4 Beta =0.08 p= 0.08  ***Total PA during mid pregnancy***  Quartile 2 Beta = 0.07 p= 0.13  Quartile 3 Beta = 0.07 p= 0.14  Quartile 4 Beta = 0.04 p= 0.35  ***Total PA during Late pregnancy***  Quartile 2 Beta = -0.04 p= 0.36  Quartile 3 Beta = -0.06 p= 0.19  Quartile 4 Beta = -0.04 p= 0.40 | CHASAN ET AL  2014  (34) |
| GWG rate as dichotomized outcome variable | | | | | |
| Achieve appropriate weekly GWG | Objective PA measure | Accumulated ≥7500 steps/d | Univariate logistic regressions | Unadjusted OR  OR=1.6 [0.38; 6.26] | COHEN T. ET AL  2009 (25) |
|  | Self’reported PA | Total average MET-hr/wk:  Accumulated ≥8.5 MET-hr/wk | Univariate logistic regressions | Unadjusted OR  **OR= 3.8 [1.18; 12.38]** | COHEN T. ET AL  2009 (25) |
| Excessive GWG rate |  | Total average MET-hr/wk categorized into 3 levels:  Low PA,  Moderate PA,  high PA | Multinomial logistic regression | Low PA  OR= 1.74 [0.77; 3.97] *  Moderate PA  OR= 1.13 [0.73; 1.76]  high PA: REF | YONG ET AL  2016  (39) |
| Inadequate GWG rate |  | Total average MET-hr/wk categorized into 3 levels:  Low PA,  Moderate PA,  high PA | Multinomial logistic regression | Low PA  OR= 0.76 [0.38;1.51]  Moderate PA  OR= 0.83 [0.54; 1.29]  high PA: REF |  |
